# Physical and Chemical Properties of Cloud Droplet Residuals and Aerosol Particles During the Arctic Ocean 2018 Expedition

**DOI:** 10.1029/2021JD036383

**Published:** 2022-06-02

**Authors:** Linn Karlsson, Andrea Baccarini, Patrick Duplessis, Darrel Baumgardner, Ian M. Brooks, Rachel Y.‐W. Chang, Lubna Dada, Kaspar R. Dällenbach, Liine Heikkinen, Radovan Krejci, W. Richard Leaitch, Caroline Leck, Daniel G. Partridge, Matthew E. Salter, Heini Wernli, Michael J. Wheeler, Julia Schmale, Paul Zieger

**Affiliations:** ^1^ Department of Environmental Science Stockholm University Stockholm Sweden; ^2^ Bolin Centre for Climate Research Stockholm University Stockholm Sweden; ^3^ Extreme Environments Research Laboratory École Polytechnique fédérale de Lausanne Sion Switzerland; ^4^ Laboratory of Atmospheric Chemistry Paul Scherrer Institute Villigen Switzerland; ^5^ Department of Physics and Atmospheric Science Dalhousie University Halifax NS Canada; ^6^ Droplet Measurement Technologies LLC Boulder CO USA; ^7^ Institute for Climate and Atmospheric Science School of Earth and Environment University of Leeds Leeds UK; ^8^ Climate Research Division Environment and Climate Change Canada Toronto ON Canada; ^9^ Department of Meteorology Stockholm University Stockholm Sweden; ^10^ College of Engineering, Mathematics and Physical Sciences University of Exeter Exeter UK; ^11^ Department of Environmental Systems Science ETH Zürich Zurich Switzerland; ^12^ Air Quality Research Division Environment and Climate Change Canada Toronto ON Canada

**Keywords:** aerosol–cloud interactions, aerosols, clouds, high Arctic, cloud residuals, in‐situ measurements

## Abstract

Detailed knowledge of the physical and chemical properties and sources of particles that form clouds is especially important in pristine areas like the Arctic, where particle concentrations are often low and observations are sparse. Here, we present in situ cloud and aerosol measurements from the central Arctic Ocean in August–September 2018 combined with air parcel source analysis. We provide direct experimental evidence that Aitken mode particles (particles with diameters ≲70 nm) significantly contribute to cloud condensation nuclei (CCN) or cloud droplet residuals, especially after the freeze‐up of the sea ice in the transition toward fall. These Aitken mode particles were associated with air that spent more time over the pack ice, while size distributions dominated by accumulation mode particles (particles with diameters ≳70 nm) showed a stronger contribution of oceanic air and slightly different source regions. This was accompanied by changes in the average chemical composition of the accumulation mode aerosol with an increased relative contribution of organic material toward fall. Addition of aerosol mass due to aqueous‐phase chemistry during in‐cloud processing was probably small over the pack ice given the fact that we observed very similar particle size distributions in both the whole‐air and cloud droplet residual data. These aerosol–cloud interaction observations provide valuable insight into the origin and physical and chemical properties of CCN over the pristine central Arctic Ocean.

## Key Points

- Aitken‐mode particles contributed significantly to cloud droplet formation in the high Arctic, especially after the transition to fall
- Residence time over the pack ice and the relative contribution of organics both increased when average particle size decreased
- Addition of aerosol mass due to aqueous‐phase chemistry was probably small over the pack ice

## 1 Introduction

The Arctic is warming faster than any other environment on the planet (Manabe & Wetherald, 1975; Serreze & Barry, 2011). The accelerated warming—Arctic amplification—leads to other changes, such as sea ice decline, glacier melt, permafrost thaw, and changes in the composition of the biological communities in the Arctic Ocean (e.g., AMAP, 2015). Aerosol particles can affect Arctic climate directly, through interactions with radiation (e.g., AMAP, 2015), and indirectly, through interactions with clouds (Albrecht, 1989; Mauritsen et al., 2011; Twomey, 1977). Our limited understanding of the feedback mechanisms and local processes related to clouds and aerosol–cloud interactions in the Arctic contributes significantly to the uncertainty in projections of future Arctic climate (IPCC, 2013). The large differences between polar night and day in terms of, for example, radiation, sea ice, cloud type and phase (liquid, mixed‐phase, or ice), and atmospheric circulation result in large seasonal variations not only in aerosol particle abundance and composition but also in their impact on clouds (e.g., Willis et al., 2018). These conditions make the Arctic environment particularly challenging to represent in large‐scale climate models (e.g., Sand et al., 2017; Schmale et al., 2021; Whaley et al., 2021).

Aerosol particles that activate into cloud droplets are called cloud condensation nuclei (CCN). There are large seasonal and regional variations in Arctic CCN number concentrations (e.g., Schmale et al., 2021; Willis et al., 2018, and references therein), and summertime high Arctic (above 80°N) concentrations are sometimes so low that either low‐level clouds do not form due to lack of CCN, or the cloud droplet number concentrations are severely limited by the available CCN (Mauritsen et al., 2011; Stevens et al., 2018). Under such CCN‐limited conditions, a small increase in the CCN concentration can lead to large changes in cloud properties and radiative effects (Mauritsen et al., 2011). If the supersaturation is sufficiently high and the concentration of accumulation mode particles is very low, there is also potential for Aitken mode aerosol particles (here particles <70 nm in diameter based on the average location of the Hoppel minimum (Hoppel et al., 1986) in our data) to act as CCN (e.g., Bulatovic et al., 2021; Leaitch et al., 2016; Lohmann & Leck, 2005).

Previous measurements in the high Arctic have suggested a range of potential sources of CCN in the summertime. In the boundary layer, the major sources are generally particles formed over the pack ice or in the marginal ice zone. This includes, for example, aerosol generated from bubble‐bursting at the surface of leads in the ice (e.g., Held et al., 2011; Leck & Bigg, 2005), blowing snow from sea ice (e.g., Frey et al., 2020; Yang et al., 2008), or secondary marine aerosol formation (e.g., Chang et al., 2011; Siegel et al., 2021) which can also include the formation of particles from inorganic vapors such as sulfuric acid (e.g., Beck et al., 2021; Leck et al., 2002) or iodic acid (e.g., Allan et al., 2015; Baccarini et al., 2020). Primary marine aerosol can be transported from outside the pack ice, but are usually a negligible source of particles to the boundary layer over the pack ice since they are efficiently scavenged by the presence of fog and low clouds at the marginal ice zone (e.g., Heintzenberg et al., 2006). Anthropogenic and other continental aerosols are generally transported above the boundary layer in summer (Stohl, 2006; Thomas et al., 2019), and some studies show that long‐range transported aerosol therefore have a limited influence on the boundary layer aerosol population (e.g., Kupiszewski et al., 2013). However, the free troposphere can be a source of aerosol particles to the boundary layer by entrainment and down‐mixing through clouds (Igel et al., 2017; Solomon et al., 2011). Therefore, it seems that the importance of free tropospheric aerosols for the formation of low‐level clouds is limited by the frequency with which these aerosols come into contact with the cloud tops (Igel et al., 2017), and the frequency of this interaction is highly uncertain.

Even in summer, low‐level clouds in the Arctic are often mixed‐phase (Shupe et al., 2006, 2013), which means that to understand them we have to understand not only CCN but also ice nucleating particles (INP) and other ice‐related cloud processes. Sources of INP outside the high Arctic include glacial dust (Tobo et al., 2019), aerosol from boreal regions (Schneider et al., 2021), and marine biogenic aerosol (Ickes et al., 2020; Wilson et al., 2015). In the summertime high Arctic, the limited influence of long‐range transport means more local sources may also be important (Bigg & Leck, 2001; Leck & Svensson, 2015). However, recently Porter et al. (2022) found that the most active INP in the central Arctic could be traced back to the waters and coasts of Siberia, while local sources in the pack ice and marginal ice zone were less important.

The high Arctic region is difficult to reach and, as a result, most measurements have been conducted in the summertime when expeditions are logistically easier. Over the last few decades, several expeditions have been carried out aboard different research vessels (e.g., Abbatt et al., 2019; Leck et al., 1996; Leck et al., 2001, 2004; Tjernström et al., 2014; Uttal et al., 2002; Wendisch et al., 2019). Detailed in situ measurement campaigns like these are necessary to deepen our process understanding of Arctic cloud formation and for improved representation of Arctic clouds in models, which are required to better understand the role that aerosol–cloud interactions play in Arctic amplification and to improve projections of climate change in the Arctic.

Here, we present measurements obtained during the Microbiology‐Ocean‐Cloud‐Coupling in the High Arctic (MOCCHA) campaign as part of the Arctic Ocean 2018 (AO18) expedition, which took place on the Swedish icebreaker (I/B) *Oden* during August and September 2018. The expedition extended from the late summer melt through the onset of the fall freeze‐up (see Vüllers et al., 2021, for a detailed meteorological overview), that is, the start of the transition from summer to fall, which allowed us to capture some of the seasonal variation in the high Arctic aerosol population and cloud properties. We set out to answer the following main research questions: (a) What are the physical and chemical properties of particles forming fog or low‐level clouds? (b) What are the main sources of cloud‐forming particles and how important are particles of local origin relative to long‐range transported aerosol? (c) How variable are the microphysical properties of cloud‐forming particles during summer and the transition to early fall?

To address these questions, a ground‐based counterflow virtual impactor (GCVI) inlet in conjunction with an interstitial and whole‐air inlet were deployed for the first time over the central Arctic Ocean. A CVI inlet samples only cloud droplets and ice crystals and allows for measurements of the aerosol particles that remain after removal of the water (termed cloud residuals), providing a direct means of characterizing the particles that are present in low‐level cloud droplets and crystals. Interstitial and total aerosol and cloud residuals were analyzed with respect to their size and concentration. Chemical composition measurements using aerosol mass spectrometry and air parcel back‐trajectory analysis were used to better understand the properties and sources of the particles. These measurements provide new insight on aerosol–cloud interactions in the high Arctic.

## 2 Methods

### 2.1 Arctic Ocean 2018 Expedition

**Figure 1:**
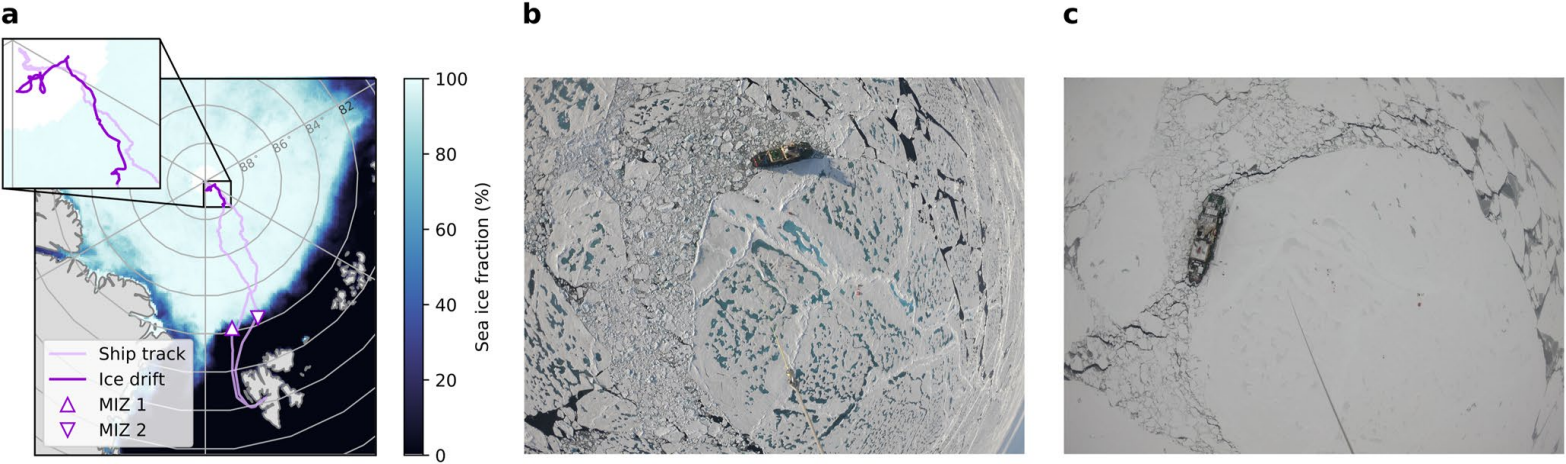
Sampling locations during the Arctic Ocean 2018 expedition. (a) The map shows the position of *Oden* throughout the expedition (light purple line). The position during the ice drift (14 August–14 September) is highlighted (dark purple) and shown in the inset. The location of a 24 hr sampling station in the marginal ice zone (MIZ) on 19 September is marked by an upside‐down triangle. The average sea ice concentration (%) for the period 2 August–20 September 2018 (Spreen et al., 2008) is also shown. To the right are photos of the ice floe (b) before (20 August) and (c) after (6 September) the freeze‐up.

Data were collected during the AO18 expedition, a joint US–Swedish research cruise to the high Arctic on board Swedish I/B *Oden*. The expedition set out from Longyearbyen, Svalbard (78.22°N, 15.65°E) on 1 August 2018 and reached the “North Pole” sampling station (89.8932°N, 38.0423°E) on 12 August 2018. On 13 August 2018, *Oden* moored to an ice floe and drifted with the pack ice for approximately 5 weeks (14 August–14 September 2018) before heading back south, reaching Longyearbyen on 22 September 2018. The ship track and the ice drift are shown in Figure 1. On the way to and from the ice drift station, two 24 hr sampling periods in the marginal ice zone (MIZ) took place on 3 August (82.1547°N, 9.9695°E) and 19 September (82.2833°N, 19.8333°E), respectively. Data from the first MIZ station are not included here because significant influence of ship exhaust was observed. The location of the second MIZ station is indicated in Figure 1.

### 2.2 Inlets and Instrumentation

Measurements of aerosols and clouds were conducted on the fourth deck of *Oden*, sampling from approximately 21 m above sea level. Inlets were mounted on the roof of container labs inside which most of the instruments were installed (see Figure S13 in Supporting Information S1 for a schematic of the set‐up and Table S5 in Supporting Information S1 for an overview of the instrumentation).

#### 2.2.1 Inlets

Three main inlets were operated during the expedition:

1. A GCVI inlet, for sampling cloud residuals (the particles that remain when cloud droplets and ice crystals are dried),
2. An interstitial inlet, for sampling non‐activated aerosol particles (<1 μm diameter), and
3. A whole‐air inlet, for sampling activated and non‐activated aerosol particles alike.

*The GCVI inlet* (Brechtel Manufacturing Inc., USA, Model 1205) uses a wind tunnel and a counterflow to separate particles based on their inertia, only letting particles above a certain size, that is, cloud droplets and ice crystals (henceforth cloud particles), enter the sample flow. A brief description will be provided below; more detailed descriptions of CVI inlets can be found in, for example, Ogren et al. (1985), Noone et al. (1988), Shingler et al. (2012). The same type of GCVI inlet used during AO18 was extensively evaluated in Karlsson et al. (2021).

The wind tunnel accelerates the cloud particles onto the inlet tip where they meet the compressed, dry air of the counterflow. This dries the cloud particles as they travel through the inlet, and the cloud residuals are subsequently sampled. The set‐up causes particle concentrations to be enhanced relative to ambient concentrations, requiring measurements downstream of the GCVI to be corrected. During AO18, the Dp_50__%_ cut size of the GCVI inlet was 7.8 ± 1.0 μm (cloud particle aerodynamic diameter), and the enrichment factor (EF) was 6.4 ± 0.2 (both cut size and EF are calculated values but agree well with experimentally determined values, see Shingler et al., 2012). The GCVI was installed on the roof of the container (inlet height approximately 20 m a.s.l.). The total sample flow was shared between the various instruments sampling behind the inlet and a bypass flow which was adjusted to maintain a constant sample flow of 15 L per minute (lpm). The GCVI was operated in manual mode, that is, it was manually turned on when there was a fog at the sampling site, determined using the GCVI weather system’s integrated visibility sensor (Belfort Instrument, USA, Model 6400). For further details on the criteria for fog/clouds, see the Supporting Information S1. The weather system also had a heated precipitation sensor, for detecting rain or snow, and an RH and temperature sensor. Our set‐up was regularly tested for potential leaks by having the GCVI turned on with the wind tunnel air speed set to 0 ms^−1^ and monitoring that the CPCs showed no particles.

The GCVI inlet does not sample all cloud particles with a sampling efficiency of unity (Karlsson et al., 2021; Shingler et al., 2012) and the actual value needs to be determined experimentally by using collocated cloud particle measurements or by comparing the accumulation mode of whole‐air and cloud residual size distribution for liquid clouds (see Karlsson et al., 2021, for more details). Due to the malfunctioning of the cloud sensor (see Supporting Information S1), no reliable size‐resolved cloud particle measurements were available for an event‐based efficiency determination. As such, a constant correction factor had to be used. The GCVI sampling efficiency determined during this study is described in more detail in the Supporting Information S1. All cloud residual concentrations presented here have been multiplied by a constant factor of 17 (after dividing by the EF), assuming cloud residual size and cloud particle size are not correlated.

*The interstitial inlet* (o.d. 12 mm, length 5 m) used a standard PM_1_ cyclone with a sharp cut, sampling 50% of the particles at 1 μm diameter. The total sample flow was approximately 16.7 lpm. The inlet was installed at a 45° angle (inlet height approximately 19 m a.s.l.). The inlet line was heated such that the relative humidity (RH) did not surpass 40% as recommended by GAW (WMO/GAW, 2016).

*The whole‐air inlet* (o.d. 3 in, length 4 m), which is designed to sample both interstitial aerosol and cloud particles up to approximately 40 μm diameter (follows guidelines for whole‐air aerosol sampling in extreme environments: Wiedensohler et al., 2014; WMO/GAW 2016), was mounted at a 45° angle on the container roof (inlet height approximately 21 m a.s.l.). The inlet was heated to approximately 40°C and had a total sample flow of 90 lpm. The RH at the end of the whole‐air inlet was also monitored (Hytelog‐USB, B + B Sensors, Germany; RH 12.1 ± 2.3% during AO18).

The inlets were fitted with valve switches, which allowed instruments to sample from the whole‐air inlet when the GCVI was not running, and also allowed some instruments to continually switch between sampling from the whole‐air inlet and the interstitial inlet (see Figure S13 and Table S5 in Supporting Information S1).

#### 2.2.2 Cloud Measurements

Cloud residual number size distributions were measured with a differential mobility particle sizer (DMPS; sample flow 1 lpm) consisting of a custom built medium Vienna type differential mobility analyzer (DMA) and a condensation particle counter (CPC; TSI Inc., USA, Model 3010). The DMPS measured particles in the size range 10–921 nm diameter. An additional CPC (TSI Inc., USA, Model 3772) was used to measure the total cloud residual number concentration. When the GCVI was not on, the DMPS instead sampled from the whole‐air inlet.

A particulate volume monitor (PVM; Gerber Scientific Inc., USA, Model PVM‐100) was also mounted on the roof of the lab container (approximately 20 m a.s.l.), measuring the liquid water content (LWC) and effective radius of the cloud particles. The PVM was regularly calibrated using the standard calibration protocol recommended by the manufacturer. Additional small drifts in the LWC and particle surface area (needed to calculate the effective radius) were corrected by subtracting the background signal during non‐cloud periods.

#### 2.2.3 Aerosol Measurements

Total aerosol particle number size distributions in the size range 10–921 nm were measured with a DMPS (sample flow 0.36 lpm) consisting of a custom built medium Vienna type DMA and a mixing condensation particle counter (MCPC; Brechtel Manufacturing Inc., USA, Model 1702) measuring behind the whole‐air inlet. An additional MCPC 1702 was used to measure the total particle concentration.

Interstitial particle concentrations were measured by a custom built scanning mobility particle sizer (SMPS; details in Supporting Information of Schmale et al., 2019), covering the size range 18–661 nm. The SMPS switched inlets every hour (even hours whole‐air inlet, odd hours interstitial inlet).

Measurements of the chemical composition of non‐refractory sub‐micron material were performed with a High Resolution Time‐of‐Flight Aerosol Mass Spectrometer (HR‐ToF‐AMS, Aerodyne Research Inc., USA) at 1 min time resolution. The AMS uses a PM_1_ aerodynamic lens; a detailed technical description can be found in DeCarlo et al. (2006). Zero measurements were conducted several times per week by installing a HEPA filter in front of the instrument inlet. These zero measurements were used to adjust the fragmentation table such that the organic CO_2_ concentration during the filter periods fluctuated around zero. The AMS measured sulfate, organics, nitrate, ammonium, and chloride with detection limits (at 1 min time resolution) of 6.51, 56.89, 0.86, 0.65, 6.20 ng m^−3^, respectively. The limit of detection (LOD) is derived from the average species concentration during the zero filter measurements plus one standard deviation (see the Supporting Information S1 for more about the LOD). In our study, methanesulfonic acid (MSA) mass concentration was calculated based on Crippa et al. (2013). The detection limit of MSA mass concentration is 0.51 ng m^−3^. The observed concentrations exceed the detection limit. A composition dependent collection efficiency (CDCE, Middlebrook et al., 2012) was applied to the AMS data. The AMS switched between the interstitial and whole‐air inlets on the same schedule as the SMPS (see above).

A multi‐angle absorption photometer (MAAP, Thermo Scientific Inc., Germany, Model 5012) was used to derive the equivalent black carbon concentration behind the whole‐air inlet. The equivalent black carbon concentration is determined by assuming a mass absorption coefficient of 6.6 m^2^ g^−1^ (see Petzold et al., 2002, for more technical details).

### 2.3 Meteorological Data

Ship position, temperature, and wind data were obtained from the navigation and meteorological systems of *Oden* (Prytherch & Tjernström, 2019). A detailed description of the meteorological conditions during AO18 can be found in Vüllers et al. (2021). Visibility and the presence of precipitation was measured by the GCVI system (see above).

### 2.4 Trajectory Analysis

Trajectories were calculated 10 days backwards from the position of the ship during the entire expedition, using the Lagrangian analysis tool LAGRANTO (Sprenger & Wernli, 2015). As meteorological input, wind fields from 3‐hourly operational ECMWF analyses that were interpolated to a regular grid with 0.5° horizontal resolution on the 137 model levels were used. In this study, we limit the trajectories to 5 days back in time as a compromise between aerosol lifetime and trajectory uncertainties (which increase with trajectory length). Trajectories were released from the five lowest model layers since we use them to explain near‐surface measurements (inlet heights around 20 m a.s.l.). Including trajectories up to model level ∼10 (average release altitude 957 hPa) does not make a significant difference in terms of source regions, so using levels 0–4 (average release altitude 1,007–1,011 hPa) should be representative of the air we sampled since our inlets were more or less at surface level. In addition, we tested the effect of restricting the trajectories to 3, 7, and 10 days (see Figure S3 in Supporting Information S1) and the source regions were similar to the ones discussed here based on 5 days.

The fractions of time each trajectory spent in the boundary layer over land, open ocean, or ice, and the time it spent above the boundary layer were calculated. The trajectory positions were interpolated to 15 min time resolution, and then each latitude–longitude point was classified as land or ocean using the Python packages Cartopy (Met Office, 2010–2015) and Shapely (Gillies et al., 2007). The sea ice concentration (SIC; %) at each data point was derived from daily average SIC from the University of Bremen satellite product (Spreen et al., 2008), and data points with a SIC >85% were classified as ice. In order to classify a trajectory point as in or above the boundary layer, the pressure along the trajectory was compared to the corresponding boundary layer height pressure from the ECMWF analyses. For points above the boundary layer no further distinction was made between land, ocean, or ice. The Arctic boundary layer is difficult to accurately represent in models (Birch et al., 2012; Graham et al., 2019; Sotiropoulou et al., 2016; Tjernström et al., 2021; Young et al., 2021). However, varying the ECMWF boundary layer pressure by ±15 hPa or assuming a constant value (965 hPa, which corresponds to the mean boundary layer pressure along all trajectory data points started from the lowest model layer) did not significantly change the results or the patterns observed (see Figure S2 in Supporting Information S1).

## 3 Results

We sampled 25 cloud events with a total of 327 measured cloud residual size distributions, corresponding to 48.8 hr of in‐cloud measurements (for more details on the definition of cloudy periods, see Supporting Information S1). The cloud events can be divided into four distinct geographical groups: the transit (icebreaking) from the MIZ to the ice drift station at the North Pole, the 5‐week ice drift period, the transit (icebreaking) back toward the MIZ, and the second MIZ station (see also Figure 1). We will begin with a data overview, then discuss the differences between the different geographical locations and later zoom in on the ice drift period which is the main focus of this study.

As AO18 took place in the summer and early fall, the clouds we sampled were liquid or mixed‐phase. Unfortunately, we had no means of distinguishing cloud phase and could therefore not characterize cloud droplet residuals and ice crystal residuals separately. Since cloud droplets usually far outnumber ice crystals in mixed‐phase clouds, it is fairly safe to assume that the vast majority of the cloud residuals we measured were cloud droplet residuals. Thus, we will use the terms cloud droplet residual or droplet residual from here on as opposed to the more general cloud residual, with the caveat that we cannot exclude the (most likely limited) influence of ice crystals on our measurements.

### 3.1 Cloud Droplet Residual Size Distributions Over the Central Arctic Ocean

**Figure 2:**
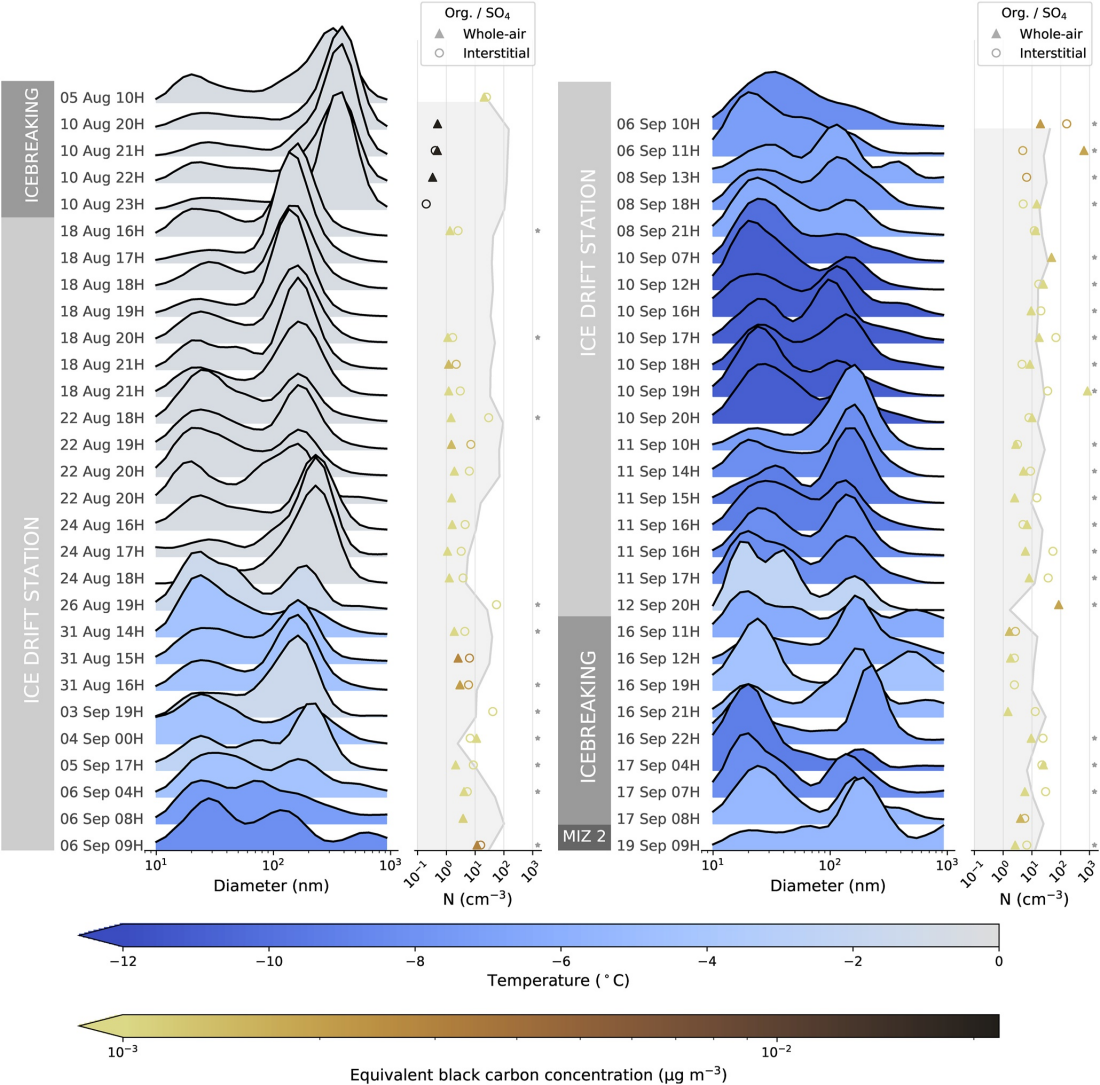
Overview of cloud droplet residual size distributions during the Arctic Ocean 2018 expedition. Normalized cloud droplet residual size distributions, color‐coded by the corresponding mean ambient temperature, are shown for all observed cloud events (each mean covers 27–72 min; longer cloud events have been split up). The size distributions have been smoothed by a five‐bin rolling average. The labels to the left of each distribution indicate the corresponding time. The shading on the axes to the right of the size distributions shows the corresponding normalization factors, that is, the droplet residual number concentrations. On the same axes, markers show the organics to sulfate ratio (measured with the Aerosol Mass Spectrometer [AMS]), color‐coded by the equivalent black carbon concentration (measured with the multi‐angle absorption photometer). Since the AMS switched between the whole‐air (△) and interstitial (◦) inlet every hour, data from both inlets are shown (depending on how individual cloud events were divided, each “hour” either includes AMS data from both the whole‐air and interstitial inlets, or only from one of the inlets). Gray asterisks mark periods where one or more AMS values were below the detection limit at the relevant time resolution.

Figure 2 shows an overview of the cloud droplet residual number size distributions measured during the AO18 expedition, in which cloud events longer than 72 min (8 DMPS scans) have been divided to show approximately hourly mean size distributions (between 27 and 72 min depending on the length of the main event). Note that the size distributions have been normalized by the integrated number concentration and smoothed with a five‐bin rolling average to better visualize the size modes. Non‐normalized size distributions are shown together with concurrent whole‐air particle size distributions in Figure S17 in Supporting Information S1. Note that the droplet residual size distributions in Figure S17 in Supporting Information S1 have been multiplied by a constant factor of 17 to account for the GCVI transmission efficiency and that this factor is an average that may over‐ or underestimate droplet residual concentrations when used on shorter timescales.

Overall, the cloud droplet residual size distributions are bimodal, but the cloud events during the first icebreaking period stand out as being very much dominated by the accumulation mode (diameter above 70 nm; >80% by number). It is clear that there are differences between the sampling locations (MIZ, icebreaking, ice drift), but Figure 2 shows that there are also differences within these groups, particularly the longer ice drift period which contains the highest number of individual cloud events (*n* = 18). The average droplet residual diameter seems to decrease with time during the expedition, increasing the relative contribution of the Aitken mode (diameter below 70 nm) from 20% at the start of the ice drift to around 70% around 10 September. However, the Aitken mode is almost always present. We can also see that the temperature was generally lower during the second half of the expedition (the freeze‐up onset was identified as 28 August in Vüllers et al., 2021). The same can be said for the number concentration of cloud droplet residuals (shaded outline on axes next to the size distributions in Figure 2), which was highest at the start of the expedition and lowest toward the end of the expedition. Note that the absolute concentration of the cloud droplet residuals is approximate (see Section 2.2 and Supporting Information S1).

The temporal trends described above are shown in more detail in Figure S18 in Supporting Information S1. The increase in Aitken mode contribution with the start of the freeze‐up of sea ice toward the end of August translates to generally smaller droplet residuals (shown as number mean diameter in Figure S18a in Supporting Information S1) and coincides with an increase of new particle formation (Baccarini et al., 2020). Figure S18 in Supporting Information S1 also shows that, although there are overarching seasonal trends in the transition from summer to fall, there can still be significant variability in the microphysical properties of the droplet residuals and whole‐air aerosol during the individual cloud events which occasionally lasted several hours.

Figure 2 also shows the organics to sulfate mass ratio (from the AMS) and the equivalent black carbon concentration (eBC; from the MAAP) associated with each time period. There is generally a slightly higher organics to sulfate ratio within the interstitial aerosol (i.e., smaller, non‐activated particles) compared to the whole‐air (all particles). This behavior could be due to aqueous‐phase production of sulfate or preferential activation of sulfate‐containing particles which are more hygroscopic than particles with relatively more organic substances, but it is difficult to say without information on the particle mixing state.

**Figure 3:**
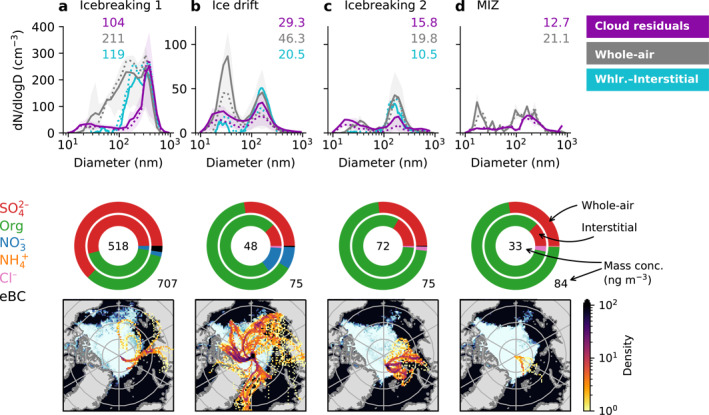
Data grouped by sampling location. (a) Transit (icebreaking) from the marginal ice zone (MIZ) to the ice drift station (4–12 August, 31 in‐cloud differential mobility particle sizer [DMPS] scans), (b) ice drift period (14 August–14 September, 250 in‐cloud DMPS scans), (c) transit (icebreaking) back toward the MIZ (15–18 September, 46 in‐cloud DMPS scans), and (d) second MIZ station (19 September, 3 in‐cloud DMPS scans). The top row shows the whole‐air particle size distributions (in gray) and the cloud droplet residual size distributions (in purple, multiplied by 17) for each period. Where available, the whole‐air minus interstitial scanning mobility particle sizer particle size distribution is also shown in blue. Note the different *y*‐axis scales (a has one scale, b–d share another). Solid and dotted lines show mean and median values, respectively, and shaded areas indicate the 25th to 75th percentile ranges. The numbers in the top right corner of each panel show the average particle number concentrations (cm^−3^). The middle row shows the chemical composition measured by the aerosol mass spectrometer and multi‐angle absorption photometer (MAAP) for each period, where the outer and inner donuts show data from the whole‐air and interstitial inlets, respectively. The numbers indicate the average total mass in ng m^−3^. Note that MAAP data are only available for the whole‐air inlet (i.e., it is only shown for the outer donut). The bottom row shows the density of five‐day backward trajectories and the average sea ice concentration for each period.

Overall, eBC concentrations were very low (2 ± 6 ng m^−3^, mean ± standard deviation, during our cloud events). Since we only have information about eBC in the whole‐air particles, it is not possible to infer anything about the eBC content of the cloud residuals. The eBC was elevated during the period before we reached the ice drift station, with a maximum concentration of approximately 24 ng m^−3^ in Figure 2 (80 ng m^−3^ at 1 min resolution). This is about an order of magnitude higher than typical eBC values over the pack ice (e.g., Leck & Persson, 1996), but such concentrations are not unheard of. Heintzenberg (1982) attributed similar observations (also in the summertime high Arctic) to an injection of polluted air from lower latitudes, and our observations also show that high eBC values are associated with a larger fraction of the trajectories having continental influence (see Figure 3a with relatively high influence from Northern Eurasia). Thus, it is possible that the higher eBC concentrations are due to transported aerosol (e.g., anthropogenic or biomass burning aerosol). However, it is also possible that there is a remaining but small influence from the ship exhaust (i.e., a diluted old ship plume), particularly during the first icebreaking period which shows the highest equivalent black carbon concentrations in addition to an increased contribution of particulate sulfate. The first icebreaking transit required some going back and forth to find a way through the pack ice, which increases the risk of encountering an old but diluted ship plume.

### 3.2 Importance of Geographic Variability

To further investigate the geographical dependence of the cloud droplet residual microphysical properties, Figure 3 shows the average cloud droplet residual size distribution for each of the periods in Figure 2, together with the corresponding average total particle size distribution. For the ice drift and icebreaking periods (Figures 3a–3c), the size distribution obtained by subtracting the average interstitial particle size distribution from the average total particle size distribution is also shown (in blue; difference is based on SMPS data). This should, ideally, be similar to the cloud droplet residual size distribution, since it is an indirect way of measuring the size distribution of activated aerosol particles. Note that the absolute concentration of the cloud droplet residuals is approximate since the data were corrected using a constant GCVI sampling efficiency (see Section 2.2 and Supporting Information S1) as opposed to using the actual measured cloud particle size distribution as done in previous work (Karlsson et al., 2021).

As already seen in Figure 2, the cloud droplet residual size distributions are bimodal on average, while which of the two modes (Aitken or accumulation) is most prominent varies. The same approximate size modes are present in the total and total‐minus‐interstitial particle size distributions; however, there are some discrepancies. For the icebreaking periods (Figures 3a and 3c), the droplet residual Aitken mode extends to slightly smaller diameters than the corresponding mode in the whole‐air data. The average difference in concentration below 25 nm is three and two particles cm^−3^ for a and c, respectively, corresponding to an excess of around 4% and 19% of the respective total cloud droplet residual concentrations. This could potentially be due to differences in the DMPS systems’ transmission efficiencies, but then it should show up in all panels. The extension of the droplet residual Aitken mode into smaller sizes than the corresponding whole‐air size mode could also be due to evaporation or volatilization losses in the GCVI or the sampling lines after it. This cannot be confirmed since no gas phase measurements were made behind the GCVI, but given that the GCVI counterflow is heated it seems plausible that some material could have evaporated. As for the total‐minus‐interstitial size distributions, they agree reasonably well with the droplet residual size distributions in the accumulation mode particle size range, while they show fewer Aitken mode particles than the droplet residual size distributions.

CVI inlet measurements can suffer from sampling artifacts such as droplet/ice crystal shattering (e.g., Karlsson et al., 2021) or particle capture by the wake effect (Pekour & Cziczo, 2011), so the question arises if the Aitken mode cloud droplet residuals are real or caused by sampling artifacts. Shattering starts to occur at Weber numbers of 10–12 (e.g., Twohy et al., 2003), which corresponds to droplet diameters of approximately 60–73 μm under our sampling conditions. Ice crystals possibly start to shatter at a slightly smaller equivalent diameters (F. Brechtel, pers. comm. 24‐02‐2022). Even so, these sizes are all significantly larger than the cloud droplets measured during AO18 (see e.g., Figure S6 in Supporting Information S1 and later Figure 5h), so droplet shattering seems an unlikely cause for the observed discrepancies. The presence of large hydrometeors like rain or drizzle increases the risk of droplet shattering producing elevated concentrations of Aitken mode residuals, but precipitation was only observed during one of the cloud events. While this event did occur during the first icebreaking period (i.e., Figure 3a), no effect on the shape of the cloud droplet residual size distribution was observed by removing the scans affected by precipitation, suggesting that this was not the cause of the observed differences.

The total‐minus‐interstitial size distributions start at 18 nm, so they cannot shed any light on what happens at smaller diameters. They do, however, show overall lower concentrations of Aitken mode particles compared to the cloud droplet residuals, as mentioned above. Part of this difference could potentially be explained by wake capture of particles in the CVI, except this type of artifact should only make up a few percent of the cloud droplet residuals at most (Pekour & Cziczo, 2011) and not an entire size mode. So what causes the difference? The SMPS (used for the total‐minus‐interstitial size distributions) has a relatively low signal‐to‐noise ratio because of the shorter scan time and higher number of bins compared to the DMPSs. This could increase the uncertainty in the total‐minus‐interstitial size distributions, particularly in the Aitken mode where the difference is expected to be small. Because the total and interstitial size distribution measurements are not concurrent, averaging does not necessarily produce better results when the size distributions are highly variable. These uncertainties might mean that the total‐minus‐interstitial and droplet residual size distributions are not necessarily inconsistent with each other. As mentioned earlier, it is also possible that evaporation or volatilization losses shrunk some droplet residuals to smaller sizes, but the extent of this effect (if it was active) is uncertain. Regardless, the fact that there is an Aitken mode (albeit smaller in concentration) in the total‐minus‐interstitial data provides independent confirmation of the existence of Aitken mode cloud droplet residuals. This is especially true for the ice drift measurements (our main focus, see Figure 3c and next section) in which there is a clear Aitken mode signal in the total‐minus‐interstitial data and the cloud droplet residual size modes line up with the total particle size modes.

The average droplet residual number concentration decreases with time, from around 100 cm^−3^ in Figure 3a to an order of magnitude lower in Figures 3b–3d, with the lowest concentrations observed during the second MIZ station (see also Figure S18c in Supporting Information S1). The total particle concentration follows a similar pattern (see also Figure S18d in Supporting Information S1). The decrease in aerosol number concentration with time is also reflected in the AMS data, which shows a decrease in the absolute mass concentrations from both the whole‐air and interstitial inlets (second row in Figure 3).

The second row in Figure 3 shows the mass fractions of sulfate (${\text{SO}}_{4}^{2-}$), organics, nitrate (${\text{NO}}_{3}^{-}$), ammonium (${\text{NH}}_{4}^{+}$), and chloride (Cl^−^) from the whole‐air (outer circle) and interstitial (inner circle) inlets, as measured by the AMS. Some compounds are only present in very small amounts, sometimes even below the detection limit (see Table S3 in Supporting Information S1 for details on signal strengths). The AMS measures non‐refractory PM_1_ and the composition measurements are applicable to the accumulation mode particles but not necessarily to the Aitken mode particles, partly because the mass is dominated by the larger particles but also because the AMS aerodynamic lens transmission efficiency is lower for Aitken mode particles (e.g., Liu et al., 2007). The AMS will also not detect all compounds that were present (e.g., compounds that do not vapourize at or below 600°C such as dust, sodium chloride or marine polymer saccharide gels (Orellana et al., 2021)). The bottom row in Figure 3 shows the density of five‐day backward trajectories for each period to further indicate potential particle source regions.

The first ice breaking period (Figure 3a) shows particles containing more sulfate than particles during the other periods and trajectories traveling along the ice edge from the Laptev Sea and from Siberia's Taymyr Peninsula. The relatively high influence of absorbing particles during this period, seen in Figure 2, is also visible in Figure 3a. The simultaneous occurrence of sulfate and black carbon in conjunction with trajectories traveling over Siberia suggests either an anthropogenic or a biomass burning influence on the aerosol population, but the sulfate could also originate from secondary marine aerosol coming from the Siberian Arctic ocean.

The back trajectories for the ice drift station (Figure 3b) and the second icebreaking and MIZ station (Figures 3c and 3d) show relatively more air circulation over the pack ice prior to sampling. A larger fraction of local, high Arctic sources is consistent with the pristine conditions indicated by the low number and mass concentrations also seen in Figures 3b–3d. The source regions do not change significantly if only trajectory points within the planetary boundary layer are considered (see Figure S4 in Supporting Information S1). The ice drift station data will be discussed in more detail in the next section.

Figure 3 shows some differences between the whole‐air and interstitial chemical compositions. For example, the whole‐air has a relatively larger sulfate fraction while the interstitial aerosol contains relatively more organics, which agrees with the general pattern in the organics to sulfate ratio seen in Figure 2. The observed differences between the whole‐air and interstitial aerosol chemical compositions are slight in comparison to the differences between the sampling periods, and the same overall trends in mass concentration and relative abundance of compounds are seen in both inlets. We therefore assume that the measured chemical compositions are also relevant for the cloud droplet residuals (at least in the accumulation mode). A simplified mass‐closure between the whole‐air size distributions and the AMS showed a reasonable agreement (see Figure S9 in Supporting Information S1), indicating that the AMS detected most of the relevant particle mass. The average droplet residual mass and composition for the periods could be estimated by taking the difference between the whole‐air and interstitial data; however, due to the fact that the whole‐air and interstitial measurements are not simultaneous, the resulting difference is uncertain. Figure S10 in Supporting Information S1 shows the result.

### 3.3 Origin and Properties of Cloud Droplet Residuals at the North Pole

**Figure 4:**
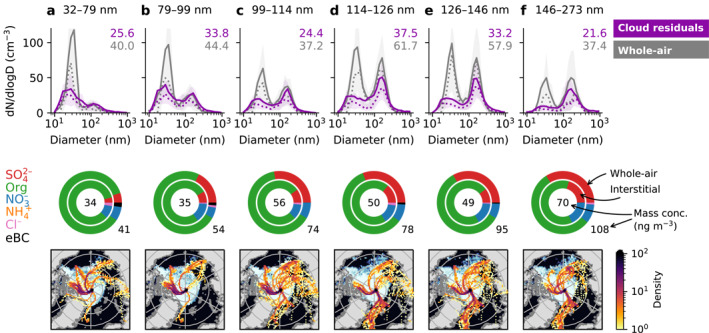
Cloud droplet residual size distributions binned by number mean diameter. Cloud droplet residual size distributions from the ice drift period (14 August–14 September 2018) were split into six equal‐size bins (41 or 42 data points) based on the number mean diameter (NMD) of each droplet residual distribution: (a) 32–79 nm, (b) 79–99 nm, (c) 99–114 nm, (d) 114–126 nm, (e) 126–146 nm, and (f) 146–273 nm. The top row shows the whole‐air particle size distributions (in gray) and the cloud droplet residual size distributions (in purple, multiplied by 17) for each bin. Solid and dotted lines show mean and median values, respectively, and shaded areas indicate the 25th to 75th percentile ranges. The numbers in the top right corner of each panel show the average particle number concentrations (cm^−3^). The middle row shows the chemical composition measured by the aerosol mass spectrometer and multi‐angle absorption photometer (MAAP) for each bin, where the outer and inner donuts show data from the whole‐air and interstitial inlets, respectively. The numbers indicate the average total mass in ng m^−3^. Note that MAAP data are only available for the whole‐air inlet (i.e., it is only shown for the outer donut). The bottom row shows the density of five‐day backward trajectories for each bin. Note that, since the trajectories were calculated for each hour and a given hour may contain size distributions belonging to more than one NMD bin, some trajectories may occur in more than one panel. The bottom row also includes the sea ice concentration for the day that contributed the most to each respective NMD bin.

The location of the start of the ice drift was chosen to be as remote as possible, with limited influence of long‐range aerosol sources, in order to study the importance of local particle sources contributing to cloud formation. Potential local sources can be of primary or secondary aerosol origin from the pack ice surface (incl. blowing snow) or from water surfaces such as leads or melt ponds. The latter ones changed significantly during the expedition, with leads around *Oden* varying in size and melt ponds starting to freeze toward the end of August. Figure 4 shows the ice drift station measurements binned by the number mean diameter (NMD) of the cloud droplet residuals. The data were split into six equal‐size groups between 32 and 273 nm NMD (each containing 41 or 42 data points). The NMD of the cloud droplet residual size distributions increases from left to right (panel a–f) in Figure 4. Like Figure 3, Figure 4 also includes information about the chemical composition and air origin.

The smaller the NMD, the larger is the Aitken mode fraction of the size distribution, and vice versa. This is also reflected in a (moderate) correlation between droplet residual NMD and whole‐air NMD (Spearman *ρ* = 0.59). During cloud measurements when the droplet residual size distributions have a larger Aitken mode than accumulation mode fraction, that is, small NMD (Figures 4a and 4b), the corresponding total particle size distributions (gray curves) are also dominated by the Aitken mode. Conversely, at larger cloud droplet residual NMD, the total particle size distributions have a relatively larger accumulation mode fraction, although the modes are more even in magnitude compared to the behavior in the droplet residuals.

Table 1 shows the parameters from fitting a bimodal lognormal distribution to the mean size distributions in Figure 4 (see also Figure S19 in Supporting Information S1 for the fit results). In the total particle size distributions, the mean modal diameter of the Aitken mode is 30–32 nm. The mean modal diameter of the accumulation mode increases from 109 nm in Figure 4a up to 163 nm in Figure 4f. These numbers are comparable to values reported from previous expeditions in the summertime high Arctic (Heintzenberg & Leck, 2012; Heintzenberg et al., 2006, and references therein). The number concentration of accumulation mode particles is generally lower for the bins with small cloud droplet residual NMD. The average total particle concentrations observed here (approximately 40–60 cm^−3^) are lower than concentrations from previous expeditions; however, the concentrations are not directly comparable since we only present data during fog periods. In fact, the whole‐air inlet used during AO18 means that our observed concentrations are likely to differ from previous measurements also during fog periods, since earlier expeditions used PM_10_ inlets which cannot sample the full cloud particle distribution (since there was a significant number of cloud droplets larger than 10 μm, see Figure S6 in Supporting Information S1). A more detailed study focusing on non‐fog measurements would be needed to determine if the aerosol population during AO18 is different from previous years.

**Table 1:** Lognormal Fit Parameters of the Mean Cloud Droplet Residual and Whole‐Air Particle Size Distributions for Each Cloud Droplet Residual Number Mean Diameter (NMD) Bin

	NMD bin (nm)	Aitken mode			Accumulation mode			Hoppel
		*N* (cm^−3^)	*d*_mod_ (nm)	*σ*	*N* (cm^−3^)	*d*_mod_ (nm)	*σ*	Min. (nm)
Residuals	32–79	15	23	1.5	11	87	2.2	81
	79–99	20	26	1.6	15	136	1.7	64
	99–114	12	27	1.8	12	153	1.4	67
	114–126	18	30	2.0	19	153	1.4	64
	126–146	13	29	1.7	19	160	1.4	67
	146–273	5	29	1.6	15	164	1.5	59
Whole‐air	32–79	33	30	1.3	5	109	1.4	64
	79–99	31	31	1.3	12	128	1.5	67
	99–114	21	31	1.4	16	145	1.5	67
	114–126	34	32	1.4	28	148	1.4	71
	126–146	30	32	1.4	26	155	1.4	71
	146–273	17	32	1.4	20	163	1.5	67

*Note.* The number concentration of the mode *N* (cm^−3^), the mean modal diameter *d*_mod_ (nm), and the geometric standard deviation *σ* are given for the Aitken mode and the accumulation mode. The locations of the Hoppel minimum between the two modes are also listed. See Figure S19 in Supporting Information S1 for the individual fits.

The average integrated droplet residual number concentration ranges between approximately 22 and 38 particles cm^−3^ in Figure 4. The mean over the ice drift period was 29 cm^−3^, and the median was 21 cm^−3^ (25th and 75th percentiles of 11 and 40 cm^−3^, respectively). As mentioned in the method section, these concentrations include a constant correction factor of 17 to account for the GCVI transmission efficiency. While this is an approximation, the cloud droplet residual concentrations are nevertheless in the range of the most frequently observed CCN concentrations during previous Arctic expeditions (also during summer and in the same area) based on CCN counter measurements for a range of different supersaturation levels (e.g., Bigg & Leck, 2001; Leck & Svensson, 2015; Mauritsen et al., 2011, and references therein). In terms of size, the fitted modal diameter of the droplet residuals varies between 23 and 30 nm for the Aitken mode. These particles are very small. Activation diameters in this size range have been indirectly observed before in the Arctic by Leaitch et al. (2016), although in their study the smallest particles were observed in higher‐altitude clouds. Modeling studies have shown that Aitken mode particles can be important CCN in clean environments with a low number concentration of accumulation mode particles (e.g., Bulatovic et al., 2021; Korhonen et al., 2008; Lohmann & Leck, 2005), which is relevant for the conditions during the ice drift period of AO18. Baccarini et al. (2020) also showed a case study from AO18 where the fog was sustained even in the absence of accumulation mode particles.

Results from a cloud parcel model (Lowe et al., 2019; Partridge et al., 2012; Roelofs & Jongen, 2004) using the fitted whole‐air size distribution from Figure 4a and assuming a uniform chemical composition are shown in Figure S16 in the Supporting Information S1. In Figure 4a, comparing the droplet residual and total particle concentrations indicates that around 65% of the aerosol particles are activated (assuming all residuals correspond to CCN). Based on the adiabatic parcel model simulations performed, this would require initializing the model with a characteristic updraft velocity of around 0.5–0.6 m s^−1^ (Figure S16b in Supporting Information S1). From the simulations we demonstrate activation of particles down to around 25 nm diameter at updraft velocities of 1 m s^−1^ for particles consisting of 30% ammonium sulfate and 70% organics (a compromise between the composition in Figure 4a and the Aitken mode chemical composition in Lawler et al., 2021, cf. below). If the particles are assumed to be more hygroscopic (in this case, pure sulfuric acid), the model predicts activation diameters approaching 20 nm in dry size (Figure S16a in Supporting Information S1). We suggest two factors that could potentially explain the high concentration of Aitken mode droplet residuals we observe: (a) A size‐dependent chemical composition (more hygroscopic Aitken mode) and (b) The role of sub‐grid scale fluctuations in updraft velocity on supersaturation, thus droplet activation (e.g., Malavelle et al., 2014; Prabhakaran et al., 2020). Larger updraft velocities that correspond to the positive tail of the updraft probability density function (PDF) which may exceed 1 m s^−1^ could be associated with activation of smaller particles.

The updraft velocities required are similar to those observed in Shupe et al. (2008) (0.42 ± 0.47 m s^−1^ mean ± SD) and Shupe et al. (2013) where Doppler cloud radar measurements of central Arctic stratus showed the tail of the updraft velocity PDF extended above 1 m s^−1^. However, the values are quite high compared to some Arctic modeling studies in which simulated updraft velocities rarely exceeded 0.5 m s^−1^ (e.g., Bulatovic et al., 2021; Sotiropoulou et al., 2020). Depending on which updrafts are most relevant for AO18, the parcel model results may not fully explain how such a large fraction of Aitken mode particles would be activated. There are many uncertainties in both the model and the measurements, and more detailed information on the in situ updraft velocities as well as size‐resolved chemical composition data (and a model that can handle such input) including information on the surface activity of organics (Lowe et al., 2019) would be needed to fully evaluate the observations. It should be noted that cloud dynamical processes such as collision‐coalescence, and entrainment were not accounted for in the adiabatic parcel model simulations. In addition, no conclusions can be drawn from these simulations regarding fog formation as supersaturation formation during radiative cooling (e.g., Poku et al., 2021) is not simulated.

In the accumulation mode, the cloud droplet residual mean modal diameter ranges from 87 nm in the smallest NMD bin up to 164 nm in the largest NMD bin (Table 1). Similarly to the total particle size distributions, the modal diameter of the droplet residual accumulation mode increases with increasing NMD and there is a tendency for the accumulation mode number concentration to be higher at larger NMD as compared to small NMD.

The average AMS mass concentration increases with increasing cloud droplet residual NMD in both the interstitial and whole‐air data (middle row in Figure 4). This reflects the increase in the accumulation mode particle concentration and size, to which the AMS is most sensitive. There is also an observable trend in the mass fractions of the individual chemical compounds with NMD—periods when small particles dominate are associated with a high fraction of organics, while the sulfate mass fraction increases with increasing particle size. However, one has to keep in mind that the AMS is sensitive to the accumulation mode and that this correlation does not necessarily imply a causation. Ammonium and chloride concentrations are, again, below the detection limit (see Table S4 in Supporting Information S1).

The interstitial data show consistently lower sulfate mass fractions than the whole‐air data. The same pattern was seen in Figure 3 and, as mentioned earlier, could be a result of aqueous‐phase production of sulfate or the activation of more hygroscopic particles with a high sulfate fraction (since cloud droplets are not sampled by the interstitial inlet). With the exception of the last NMD bin, the relative difference between the sulfate fractions in the two inlets becomes larger with larger NMD. Figure S11 in Supporting Information S1 shows the difference between the whole‐air and interstitial data as a proxy for the droplet residual mass and composition. While the trend is not quite as linear as in the whole‐air data, Figure S11 in Supporting Information S1 shows that the cloud droplet residuals in the first two NMD groups have, on average, a higher fraction of organics (more than half the mass) than the last four NMD groups (in which sulfate makes up more than half the mass). This is in qualitative agreement with recent results from Antarctica (Saliba et al., 2021; Sanchez et al., 2021; Twohy et al., 2021), although it should be noted that the meteorology and aerosol sources might differ between the two polar regions. However, as demonstrated earlier, this approximation of the droplet residual composition using the AMS data is quite uncertain. As far as the Aitken mode is concerned, Lawler et al. (2021) determined the mean Aitken mode composition during fog periods (during AO18) to include at least 25% organics such as secondary organic aerosol and polysaccharides, while another 25% consisted of inorganic compounds (mostly sulfate). Approximately half of the mass was not determined in Lawler et al. (2021), so it is difficult to say whether the Aitken mode particles consist of mostly organic or inorganic material.

There is an effect of source regions on the size and composition of the cloud droplet residuals—the groups with smallest NMD (Figures 4a and 4b) have more air influence from the pack ice near the Canadian Arctic, while the groups with larger NMD (Figures 4d–4f) are relatively more influenced by air coming from the Greenland Sea and Siberia. More trajectories traveling over continents or open water, or indeed the MIZ, is consistent with the higher sulfate fractions observed for these particles. Figure 4c is a mix of the other two groups, with influence from all aforementioned source regions. The source regions do not change significantly if only trajectory points within the planetary boundary layer are considered (see Figure S5 in Supporting Information S1).

**Figure 5:**
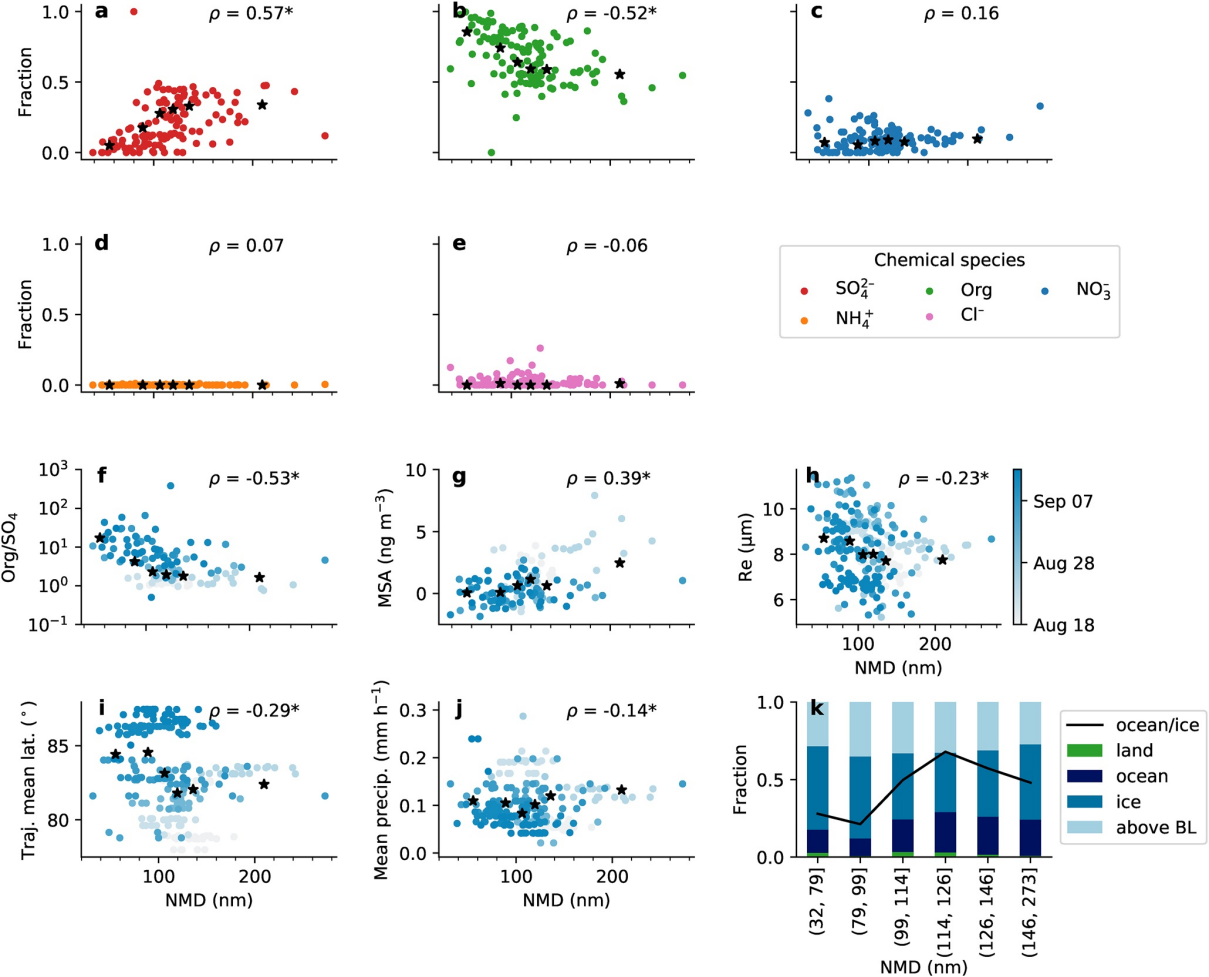
(a–i) scatter plots of cloud droplet residual number mean diameter (NMD) versus (a–e) whole‐air mass fractions of sulfate, organics, nitrate, ammonium, and chloride, (f) whole‐air organics to sulfate mass ratio, (g) whole‐air methanesulfonic acid concentration, (h) cloud droplet effective radius (from particulate volume monitor), (i) back trajectory mean latitude, (j) average precipitation rate along the trajectories. In (a–j), Spearman *ρ* correlation coessfficients are included, with an asterisk if the *p*‐value < 0.05. The black stars show the mean values for each NMD bin. In (f–j), the data points are color coded by the day of year. Panel (k) shows a stacked bar plot of the back trajectory time fraction spent over land, ocean, ice (sea ice concentration >85%), and above the boundary layer for each NMD bin. The black line shows the time fraction spent over ocean divided by the time fraction spent over ice.

The trends described above are studied in more detail in Figure 5. The figure shows scatter plots and corresponding correlation coefficients for different chemical and trajectory parameters versus cloud droplet residual NMD. The (whole‐air) sulfate mass fraction increases with increasing NMD (Figure 5a), while the organic fraction decreases (Figure 5b) (Spearman *ρ* values of 0.57 and −0.52 based on whole‐air AMS data for individual DMPS scans). There is also a positive correlation between droplet residual NMD and MSA concentration derived from the AMS measurements (Figure 5g). Similar trends are observed in the interstitial data, but with slightly weaker correlation coefficients (not shown). It is tempting to use these correlations to infer the chemical composition of the Aitken mode; however, even in the interstitial data, the Aitken mode makes up less than 10% of the total volume (i.e., mass) of the size distributions. Therefore, the differences in composition between the NMD groups probably only reflect differences in the accumulation mode composition, which, in turn, could be related to source regions or atmospheric processing of the aerosol particles. However, if the aerosol population is not externally mixed or if both the Aitken and accumulation mode particles were growing by condensation, the chemical composition measurements will also be relevant for the Aitken mode.

In the absence of fog or clouds, the primary sink for MSA and sulfuric acid vapors is condensation onto previously existing aerosol particles (Kerminen & Leck, 2001) or dry deposition (Baccarini et al., 2020). If fog or clouds are present, sulfate mass can also be added to the particles through aqueous‐phase production (aqueous oxidation of sulfur dioxide and dimethyl sulfide) or direct water uptake of MSA and sulfuric acid vapors, though the latter is probably less significant (e.g., Barnes et al., 2006; Hoffmann et al., 2016; Chen et al., 2018). In‐cloud production of sulfate can produce bimodal size distributions like the ones we observe. Table 1, which shows the lognormal fit parameters of the mean distributions in Figure 4, also includes the approximate locations of the Hoppel minimum, which, if formed through cloud processing, is the separation of processed (activated) and non‐processed aerosol particles (Hoppel et al., 1986).

The geometric standard deviation of the accumulation mode is almost constant at 1.4–1.5 for both cloud residuals and whole‐air (apart from the first two residual classes for which the standard deviation is slightly larger with 1.7 and 2.2). The Hoppel minima of all binned size distributions from Figure 4 range from 64 to 71 nm for the whole‐air measurements, which is in the same range as in previous expeditions (Heintzenberg & Leck, 2012) and measurements from Alert, Canada (Leaitch et al., 2013). The corresponding Hoppel minima of the cloud droplet residuals lie in the same range (59–81 nm, see Table 1). It is notable that we observe cloud droplet residuals smaller than the Hoppel minima indicated by the whole‐air measurements and that similar Hoppel minima are observed in the cloud droplet residual size distributions. While the low number of accumulation mode particles (5–28 cm^−3^, Table 1) could help the Aitken mode particles take up water and activate into cloud droplets, our measurements suggest that no significant extra mass was added during cloud processing over the pack ice—otherwise, the activated Aitken mode particles would grow and the minimum would move, yet we observe a fairly constant minimum at 65–70 nm in both the whole‐air and cloud droplet residual size distributions—and that the characteristics of the accumulation mode particles transported to the North Pole were determined by the sources and cloud activation outside the pack ice. In other words, while there may be cloud processing and addition of secondary mass in, for example, the marginal ice zone, our observations suggest that additional cloud encounters as the air mass travels over the pack ice do not lead to any significant further additions of mass.

Figure 5k, which shows the fraction of time the trajectories spent over land, ocean, ice, and above the boundary layer for each NMD bin, also shows that the time fraction over ocean relative to ice increases with increasing NMD. Relating this back to the trends in chemical composition, it would seem that a higher fraction of organic material in the accumulation mode is associated with more travel time over the ice, while a higher sulfate fraction is associated with more travel time over the ocean. Given the apparent lack of aqueous‐phase production of sulfate over the pack ice, it is a possibility that the differences in chemical composition mostly reflect different stages of aging of the accumulation mode aerosol particles. However, the source regions outside the pack ice also differ between the NMD groups (as seen in Figure 4)—the Laptev and East Siberian Seas dominate for the smaller NMD groups, while the Greenland Sea dominates for the larger NMD groups. This, in combination with the fact that there is also some covariance with time (smaller NMD is more common after the freeze‐up/the transition to fall, see colors in Figures 5f–5j and also Figures 2, S16 in Supporting Information S1) could also mean that the differences in chemical composition are simply a result of different source regions with different environmental conditions. For example, they could be related to seasonal differences in the biological activity in the ocean and ice. The back trajectories for the size distributions with larger NMD also, on average, tend to originate from further away (Figure 5i), that is, closer to potential anthropogenic sources of sulfate and/or sulfur dioxide (besides the oceanic sulfur sources).

Trajectories that have traveled from far away have, on average, spent relatively less time over the pack ice before reaching the sampling location and might therefore have relatively less contact with local aerosol sources. Trajectory analyses from previous expeditions have also shown correlations between travel time and particle size modes, indicating sources of small particles in the inner Arctic (Heintzenberg et al., 2006, 2015). The dominance of the Aitken mode could potentially also be a result of more removal of the larger particles during transport; however, only a very weak anti‐correlation between particle NMD and average precipitation rate along the trajectories was observed (Figure 5j). In summary, we see that cloud particles originating from air that spent more time over the pack ice are on average smaller compared to larger cloud seeds which have more oceanic influence. The relationship remains the same if the calculations of surface residence times are done with 7‐ or 10‐day back‐trajectories (Figure S3 in Supporting Information S1), since increasing the trajectory length mainly adds more points in the free troposphere.

## 4 Conclusions

Within this work, we provide direct and extensive experimental evidence that cloud‐forming particles over the central Arctic Ocean are strongly influenced by Aitken mode particles with an average mode diameter of around 30 nm. The expedition covered the end of the high‐Arctic summer season and the transition into fall, allowing us to capture the freeze‐up period and some of the seasonality in the aerosol and cloud droplet residual populations.

Seasonal variation in the cloud droplet residual number concentration, size, and Aitken mode fraction were observed. Overall, the whole‐air and droplet residual particle concentrations and their size decreased toward fall, while the Aitken mode fraction of the cloud droplet residual size distributions increased, especially after the start of the freeze‐up of the sea ice. However, the variability within individual cloud events was sometimes very high. During the ice drift period, the average cloud droplet residual number concentrations were in the range of 20–30 particles cm^−3^, which is comparable to CCN measurement results from previous expeditions in the same region and time of year. The Aitken mode was almost always present in the droplet residual size distributions, strongly supporting previous indirect measurements and model predictions that the Aitken mode may contribute significantly to cloud droplet formation in remote regions when accumulation mode particle concentrations are low.

Cloud droplet residual size distributions with a dominance of smaller particles were associated with trajectories that traveled for longer time periods over the pack ice compared to size distributions dominated by larger particles, which were associated with trajectories that had more influence from the MIZ and the open ocean. However, co‐variance with time and chemistry made it difficult to disentangle the seasonal component from the source analysis. The size of cloud droplet residuals showed a clear correlation with the sulfate and organic mass fraction, with organics being more dominant for smaller modal diameters of the accumulation mode and sulfate dominating when the modal diameter of the accumulation mode was larger. Addition of aerosol mass to the accumulation mode due to aqueous‐phase chemistry and cloud processing over the pack ice was probably small due to almost unchanged Hoppel minima and the presence of cloud droplet residuals smaller than these minima. This suggests that particles with a large sulfate contribution most likely had their source in the MIZ or beyond and were transported over the pack ice to the measurement site.

For the first time, a ground‐based virtual impactor inlet was used over the central Arctic Ocean for an extended period allowing new perspectives in understanding the characteristics of cloud‐forming particles and their role in this pristine environment. Future work should shed further light on the details of the particle and cloud residual chemical composition and should further disentangle the influence of meteorology and other cloud properties (including cloud phase and cloud layering) on the in situ measured cloud residual properties.

## Supporting information

Supporting Information S1

## Data Availability

The data of the Arctic Ocean 2018 expedition is available at the data centre of the Bolin Centre for Climate Research (https://bolin.su.se/data/oden-ao-2018-expedition-1; Leck et al., 2021). The main data used within this study is found here: https://doi.org/10.17043/oden-ao-2018-aerosol-cvi-1 (Karlsson et al., 2022).

## References

[jgrd57978-bib-0001] Abbatt, J. P. , Leaitch, W. R. , Aliabadi, A. A. , Bertram, A. K. , Blanchet, J. P. , Boivin‐Rioux, A. , et al. (2019). Overview paper: New insights into aerosol and climate in the Arctic. Atmospheric Chemistry and Physics, 19(4), 2527–2560. 10.5194/acp-19-2527-2019

[jgrd57978-bib-0002] Albrecht, B. A. (1989). Aerosols, cloud microphysics, and fractional cloudiness. Science, 245(4923), 1227–1230. 10.1126/science.245.4923.1227 17747885

[jgrd57978-bib-0003] Allan, J. D. , Williams, P. I. , Najera, J. , Whitehead, J. D. , Flynn, M. J. , Taylor, J. W. , et al. (2015). Iodine observed in new particle formation events in the Arctic atmosphere during ACCACIA. Atmospheric Chemistry and Physics, 15(10), 5599–5609. 10.5194/acp-15-5599-2015

[jgrd57978-bib-0004] AMAP. (2015). AMAP Assessment 2015: Black carbon and ozone as Arctic climate forcers. Arctic Monitoring and Assessment Programme (AMAP).

[jgrd57978-bib-0005] Baccarini, A. , Karlsson, L. , Dommen, J. , Duplessis, P. , Vüllers, J. , Brooks, I. M. , et al. (2020). Frequent new particle formation over the high Arctic pack ice by enhanced iodine emissions. Nature Communications, 11(1), 4924. 10.1038/s41467-020-18551-0 PMC7529815 33004812

[jgrd57978-bib-0006] Barnes, I. , Hjorth, J. , & Mihalopoulos, N. (2006). Dimethyl sulfide and dimethyl sulfoxide and their oxidation in the atmosphere. Chemical Reviews, 106(3), 940–975. (PMID: 16522014). 10.1021/cr020529+ 16522014

[jgrd57978-bib-0007] Beck, L. J. , Sarnela, N. , Junninen, H. , Hoppe, C. J. M. , Garmash, O. , Bianchi, F. , et al. (2021). Differing mechanisms of new particle formation at two Arctic sites. Geophysical Research Letters, 48(4), 1–11. 10.1029/2020GL091334

[jgrd57978-bib-0008] Bigg, E. K. , & Leck, C. (2001). Cloud‐active particles over the central Arctic ocean. Journal of Geophysical Research, 106(D23), 32155–32166. 10.1029/1999JD901152

[jgrd57978-bib-0009] Birch, C. E. , Brooks, I. M. , Tjernström, M. , Shupe, M. D. , Mauritsen, T. , Sedlar, J. , et al. (2012). Modelling atmospheric structure, cloud and their response to CCN in the central Arctic: ASCOS case studies. Atmospheric Chemistry and Physics, 12(7), 3419–3435. 10.5194/acp-12-3419-2012

[jgrd57978-bib-0010] Bulatovic, I. , Igel, A. L. , Leck, C. , Heintzenberg, J. , Riipinen, I. , & Ekman, A. M. L. (2021). The importance of Aitken mode aerosol particles for cloud sustenance in the summertime high Arctic—A simulation study supported by observational data. Atmospheric Chemistry and Physics, 21(5), 3871–3897. 10.5194/acp-21-3871-2021

[jgrd57978-bib-0011] Chang, R. Y. , Leck, C. , Graus, M. , Müller, M. , Paatero, J. , Burkhart, J. F. , et al. (2011). Aerosol composition and sources in the central Arctic Ocean during ASCOS. Atmospheric Chemistry and Physics, 11(20), 10619–10636. 10.5194/acp-11-10619-2011

[jgrd57978-bib-0012] Chen, Q. , Sherwen, T. , Evans, M. , & Alexander, B. (2018). DMS oxidation and sulfur aerosol formation in the marine troposphere: A focus on reactive halogen and multiphase chemistry. Atmospheric Chemistry and Physics, 18(18), 13617–13637. 10.5194/acp-18-13617-2018

[jgrd57978-bib-0013] Crippa, M. , El Haddad, I. , Slowik, J. G. , De Carlo, P. F. , Mohr, C. , Heringa, M. F. , et al. (2013). Identification of marine and continental aerosol sources in Paris using high resolution aerosol mass spectrometry. Journal of Geophysical Research: Atmospheres, 118(4), 1950–1963. 10.1002/jgrd.50151

[jgrd57978-bib-0014] DeCarlo, P. F. , Kimmel, J. R. , Trimborn, A. , Northway, M. J. , Jayne, J. T. , Aiken, A. C. , et al. (2006). Field‐deployable, high‐resolution, time‐of‐flight aerosol mass spectrometer. Analytical Chemistry, 78(24), 8281–8289. (PMID: 17165817). 10.1021/ac061249n 17165817

[jgrd57978-bib-0015] Frey, M. M. , Norris, S. J. , Brooks, I. M. , Anderson, P. S. , Nishimura, K. , Yang, X. , et al. (2020). First direct observation of sea salt aerosol production from blowing snow above sea ice. Atmospheric Chemistry and Physics, 20(4), 2549–2578. 10.5194/acp-20-2549-2020

[jgrd57978-bib-0016] Gillies, S. , Bierbaum, A. , Lautaportti, K. , & Tonnhofer, O. (2007). Shapely: Manipulation and analysis of geometric objects. Retrieved from https://github.com/Toblerity/Shapely

[jgrd57978-bib-0017] Graham, R. M. , Hudson, S. R. , & Maturilli, M. (2019). Improved performance of ERA5 in Arctic gateway relative to four global atmospheric reanalyses. Geophysical Research Letters, 46(11), 6138–6147. 10.1029/2019GL082781

[jgrd57978-bib-0018] Heintzenberg, J. (1982). Size‐segregated measurements of particulate elemental carbon and aerosol light absorption at remote Arctic locations. Atmospheric Environment, 16(10), 2461–2469. 10.1016/0004-6981(82)90136-6

[jgrd57978-bib-0019] Heintzenberg, J. , & Leck, C. (2012). The summer aerosol in the central Arctic 1991–2008: Did it change or not? Atmospheric Chemistry and Physics, 12(9), 3969–3983. 10.5194/acp-12-3969-2012

[jgrd57978-bib-0020] Heintzenberg, J. , Leck, C. , Birmili, W. , Wehner, B. , Tjernström, M. , & Wiedensohler, A. (2006). Aerosol number‐size distributions during clear and fog periods in the summer high Arctic: 1991, 1996 and 2001. Tellus Series B: Chemical and Physical Meteorology, 58(1), 41–50. 10.1111/j.1600-0889.2005.00171.x

[jgrd57978-bib-0021] Heintzenberg, J. , Leck, C. , & Tunved, P. (2015). Potential source regions and processes of aerosol in the summer Arctic. Atmospheric Chemistry and Physics, 15(11), 6487–6502. 10.5194/acp-15-6487-2015

[jgrd57978-bib-0022] Held, A. , Brooks, I. M. , Leck, C. , & Tjernström, M. (2011). On the potential contribution of open lead particle emissions to the central Arctic aerosol concentration. Atmospheric Chemistry and Physics, 11(7), 3093–3105. 10.5194/acp-11-3093-2011

[jgrd57978-bib-0023] Hoffmann, E. H. , Tilgner, A. , Schrödner, R. , Bräuer, P. , Wolke, R. , & Herrmann, H. (2016). An advanced modeling study on the impacts and atmospheric implications of multiphase dimethyl sulfide chemistry. Proceedings of the National Academy of Sciences, 113(42), 11776–11781. 10.1073/pnas.1606320113 PMC5081572 27688763

[jgrd57978-bib-0024] Hoppel, W. , Frick, G. , & Larson, R. (1986). Effect of nonprecipitating clouds on the aerosol size distribution in the marine boundary layer. Geophysical Research Letters, 13(2), 125–128. 10.1029/gl013i002p00125

[jgrd57978-bib-0025] Ickes, L. , Porter, G. C. E. , Wagner, R. , Adams, M. P. , Bierbauer, S. , Bertram, A. K. , et al. (2020). The ice‐nucleating activity of Arctic sea surface microlayer samples and marine algal cultures. Atmospheric Chemistry and Physics, 20(18), 11089–11117. 10.5194/acp-20-11089-2020

[jgrd57978-bib-0026] Igel, A. L. , Ekman, A. M. L. , Leck, C. , Tjernström, M. , Savre, J. , & Sedlar, J. (2017). The free troposphere as a potential source of Arctic boundary layer aerosol particles. Geophysical Research Letters, 44(13), 7053–7060. 10.1002/2017GL073808

[jgrd57978-bib-0027] IPCC . (2013). Climate change 2013: The physical science basis. In T. Stocker , D. Qin , G. K. Plattner , L. Alexander , S. Allen , N. Bindoff , et al. (Ed.), Working group I contribution of to the fifth assessment report of the intergovernmental panel on climate change. Cambridge University Press.

[jgrd57978-bib-0028] Karlsson, L. , Krejci, R. , Koike, M. , Ebell, K. , & Zieger, P. (2021). A long‐term study of cloud residuals from low‐level Arctic clouds. Atmospheric Chemistry and Physics, 21(11), 8933–8959. 10.5194/acp-21-8933-2021

[jgrd57978-bib-0029] Karlsson, L. , Zieger, P. , Salter, M. , Dada, L. , Schmale, J. , Dällenbach, K. , & Baccarini, A. (2022). Cloud droplet residual and aerosol measurements from the Arctic Ocean 2018 expedition. Bolin Centre Database [Dataset]. Dataset version 1. 10.17043/oden-ao-2018-aerosol-cvi-1

[jgrd57978-bib-0030] Kerminen, V.‐M. , & Leck, C. (2001). Sulfur chemistry over the central Arctic Ocean during the summer: Gas‐to‐particle transformation. Journal of Geophysical Research, 106(D23), 32087–32099. 10.1029/2000JD900604

[jgrd57978-bib-0031] Korhonen, H. , Carslaw, K. S. , Spracklen, D. V. , Riley, D. A. , & Ström, J. (2008). A global model study of processes controlling aerosol size distributions in the Arctic spring and summer. Journal of Geophysical Research, 113(8), 1–20. 10.1029/2007JD009114

[jgrd57978-bib-0032] Kupiszewski, P. , Leck, C. , Tjernström, M. , Sjogren, S. , Sedlar, J. , Graus, M. , et al. (2013). Vertical profiling of aerosol particles and trace gases over the central Arctic Ocean during summer. Atmospheric Chemistry and Physics, 13(24), 12405–12431. 10.5194/acp-13-12405-2013

[jgrd57978-bib-0033] Lawler, M. J. , Saltzman, E. S. , Karlsson, L. , Zieger, P. , Salter, M. , Baccarini, A. , et al. (2021). New insights into the composition and origins of ultrafine aerosol in the summertime high Arctic. Geophysical Research Letters, 48(21), 1–11. 10.1029/2021GL094395

[jgrd57978-bib-0034] Leaitch, W. R. , Korolev, A. , Aliabadi, A. A. , Burkart, J. , Willis, M. D. , Abbatt, J. P. , et al. (2016). Effects of 20‐100 nm particles on liquid clouds in the clean summertime Arctic. Atmospheric Chemistry and Physics, 16(17), 11107–11124. 10.5194/acp-16-11107-2016

[jgrd57978-bib-0035] Leaitch, W. R. , Sharma, S. , Huang, L. , Toom‐Sauntry, D. , Chivulescu, A. , Macdonald, A. M. , et al. (2013). Dimethyl sulfide control of the clean summertime Arctic aerosol and cloud. Elementa: Science of the Anthropocene, 1, 1–12. 10.12952/journal.elementa.000017

[jgrd57978-bib-0036] Leck, C. , & Bigg, E. K. (2005). Biogenic particles in the surface microlayer and overlaying atmosphere in the central Arctic Ocean during summer. Tellus B: Chemical and Physical Meteorology, 57(4), 305–316. 10.3402/tellusb.v57i4.16546

[jgrd57978-bib-0037] Leck, C. , Bigg, E. K. , Covert, D. S. , Heintzenberg, J. , Maenhaut, W. , Nilsson, E. D. , & Wiedensohler, A. (1996). Overview of the atmospheric research program during the International Arctic Ocean Expedition of 1991 (IAOE‐91) and its scientific results. Tellus B: Chemical and Physical Meteorology, 48(2), 136–155. 10.3402/tellusb.v48i2.15833

[jgrd57978-bib-0038] Leck, C. , Matrai, P. , Achtert, P. , Adams, M. , Baccarini, A. , Brooks, B. , et al. (2021). Data from expedition Arctic ocean, 2018. Dataset version 1. Bolin Centre Database [Dataset]. 10.17043/oden-ao-2018-expedition-1

[jgrd57978-bib-0039] Leck, C. , Nilsson, E. D. , Bigg, E. K. , & Bäcklin, L. (2001). Atmospheric program on the Arctic Ocean Expedition 1996 (AOE‐96): An overview of scientific goals, experimental approach, and instruments. Journal of Geophysical Research, 106(D23), 32051–32067. 10.1029/2000JD900461

[jgrd57978-bib-0040] Leck, C. , Norman, M. , & Bigg, E. K. (2002). Chemical composition and sources of the high Arctic aerosol relevant for cloud formation. Journal of Geophysical Research, 107(D12), 4135. 10.1029/2001JD001463

[jgrd57978-bib-0041] Leck, C. , & Persson, C. (1996). Seasonal and short‐term variability in dimethyl sulfide, sulfur dioxide and biogenic sulfur and sea salt aerosol particles in the Arctic marine boundary layer during summer and autumn. Tellus B: Chemical and Physical Meteorology, 48(2), 272–299. 10.3402/tellusb.v48i2.15891

[jgrd57978-bib-0042] Leck, C. , & Svensson, E. (2015). Importance of aerosol composition and mixing state for cloud droplet activation over the Arctic pack ice in summer. Atmospheric Chemistry and Physics, 15(5), 2545–2568. 10.5194/acp-15-2545-2015

[jgrd57978-bib-0043] Leck, C. , Tjernström, M. , Matrai, P. , Swietlicki, E. , & Bigg, K. (2004). Can marine micro‐organisms influence melting of the Arctic pack ice? EOS, Transactions American Geophysical Union, 85(3), 25. 10.1029/2004EO030001

[jgrd57978-bib-0044] Liu, P. S. K. , Deng, R. , Smith, K. A. , Williams, L. R. , Jayne, J. T. , Canagaratna, M. R. , et al. (2007). Transmission efficiency of an aerodynamic focusing lens system: Comparison of model calculations and laboratory measurements for the aerodyne aerosol mass spectrometer. Aerosol Science and Technology, 41(8), 721–733. 10.1080/02786820701422278

[jgrd57978-bib-0045] Lohmann, U. , & Leck, C. (2005). Importance of submicron surface‐active organic aerosols for pristine Arctic clouds. Tellus B: Chemical and Physical Meteorology, 57(3), 261–268. 10.1111/j.1600-0889.2005.00144.x

[jgrd57978-bib-0046] Lowe, S. J. , Partridge, D. G. , Davies, J. F. , Wilson, K. R. , Topping, D. , & Riipinen, I. (2019). Key drivers of cloud response to surface‐active organics. Nature Communications, 10(1), 5214. 10.1038/s41467-019-12982-0 PMC6861266 31740670

[jgrd57978-bib-0047] Malavelle, F. F. , Haywood, J. M. , Field, P. R. , Hill, A. A. , Abel, S. J. , Lock, A. P. , et al. (2014). A method to represent subgrid‐scale updraft velocity in kilometer‐scale models: Implication for aerosol activation. Journal of Geophysical Research: Atmospheres, 119(7), 4149–4173. 10.1002/2013JD021218

[jgrd57978-bib-0048] Manabe, S. , & Wetherald, R. T. (1975). The effects of doubling the CO_2_ concentration on the climate of a general circulation model. Journal of the Atmospheric Sciences, 32(1), 3–15. 10.1175/1520-0469(1975)032<0003:TEODTC>2.0.CO;2

[jgrd57978-bib-0049] Mauritsen, T. , Sedlar, J. , Tjernström, M. , Leck, C. , Martin, M. , Shupe, M. , et al. (2011). An Arctic CCN‐limited cloud‐aerosol regime. Atmospheric Chemistry and Physics, 11(1), 165–173. 10.5194/acp-11-165-2011

[jgrd57978-bib-0050] Met Office . (2010‐2015). Cartopy: A cartographic python library with a matplotlib interface [Computer software manual]. Exeter, Devon. Retrieved from http://scitools.org.uk/cartopy

[jgrd57978-bib-0051] Middlebrook, A. M. , Bahreini, R. , Jimenez, J. L. , & Canagaratna, M. R. (2012). Evaluation of composition‐dependent collection efficiencies for the aerodyne aerosol mass spectrometer using field data. Aerosol Science and Technology, 46(3), 258–271. 10.1080/02786826.2011.620041

[jgrd57978-bib-0052] Noone, K. J. , Ogren, J. A. , Heintzenberg, J. , Charlson, R. J. , & Covert, D. S. (1988). Design and calibration of a counterflow virtual impactor for sampling of atmospheric fog and cloud droplets. Aerosol Science and Technology, 8(3), 235–244. 10.1080/02786828808959186

[jgrd57978-bib-0053] Ogren, J. A. , Heintzenberg, J. , & Charlson, R. J. (1985). In‐situ sampling of clouds with a droplet to aerosol converter. Geophysical Research Letters, 12(3), 121–124. 10.1029/GL012i003p00121

[jgrd57978-bib-0054] Orellana, M. V. , Hansell, D. A. , Matrai, P. A. , & Leck, C. (2021). Marine polymer‐gels’ relevance in the atmosphere as aerosols and CCN. Gels, 7(4), 185. 10.3390/gels7040185 34842644 PMC8628772

[jgrd57978-bib-0055] Partridge, D. G. , Vrugt, J. A. , Tunved, P. , Ekman, A. M. L. , Struthers, H. , & Sorooshian, A. (2012). Inverse modelling of cloud‐aerosol interactions—Part 2: Sensitivity tests on liquid phase clouds using a Markov chain Monte Carlo based simulation approach. Atmospheric Chemistry and Physics, 12(6), 2823–2847. 10.5194/acp-12-2823-2012

[jgrd57978-bib-0056] Pekour, M. S. , & Cziczo, D. J. (2011). Wake capture, particle breakup, and other artifacts associated with counterflow virtual impaction. Aerosol Science and Technology, 45(6), 748–754. 10.1080/02786826.2011.558942

[jgrd57978-bib-0057] Petzold, A. , Kramer, H. , & Schönlinner, M. (2002). Continuous measurement of atmospheric black carbon using a multi‐angle absorption photometer. Environmental Science & Pollution Research, Special Issue, 4, 78–82.

[jgrd57978-bib-0058] Poku, C. , Ross, A. N. , Hill, A. A. , Blyth, A. M. , & Shipway, B. (2021). Is a more physical representation of aerosol activation needed for simulations of fog? Atmospheric Chemistry and Physics, 21(9), 7271–7292. 10.5194/acp-21-7271-2021

[jgrd57978-bib-0060] Porter, G. C. E. , Adams, M. P. , Brooks, I. M. , Ickes, L. , Karlsson, L. , Leck, C. , et al. (2022). Highly active ice‐nucleating particles at the summer North Pole. Journal of Geophysical Research: Atmospheres, 127(6), e2021JD036059. 10.1029/2021JD036059 PMC9285974 35865411

[jgrd57978-bib-0061] Prabhakaran, P. , Shawon, A. S. M. , Kinney, G. , Thomas, S. , Cantrell, W. , & Shaw, R. A. (2020). The role of turbulent fluctuations in aerosol activation and cloud formation. Proceedings of the National Academy of Sciences, 117(29), 16831–16838. 10.1073/pnas.2006426117 PMC7382222 32641512

[jgrd57978-bib-0062] Prytherch, J. , & Tjernström, M. (2019). Navigation, meteorological and surface seawater data from the Arctic Ocean 2018 expedition. Dataset version 1.0. Bolin Centre Database. 10.17043/ao2018-navigation

[jgrd57978-bib-0063] Saliba, G. , Sanchez, K. J. , Russell, L. M. , Twohy, C. H. , Roberts, G. C. , Lewis, S. , et al. (2021). Organic composition of three different size ranges of aerosol particles over the Southern Ocean. Aerosol Science and Technology, 55(3), 268–288. 10.1080/02786826.2020.1845296

[jgrd57978-bib-0064] Sanchez, K. J. , Roberts, G. C. , Saliba, G. , Russell, L. M. , Twohy, C. , Reeves, J. M. , et al. (2021). Measurement report: Cloud processes and the transport of biological emissions affect southern ocean particle and cloud condensation nuclei concentrations. Atmospheric Chemistry and Physics, 21(5), 3427–3446. 10.5194/acp-21-3427-2021

[jgrd57978-bib-0065] Sand, M. , Samset, B. H. , Balkanski, Y. , Bauer, S. , Bellouin, N. , Berntsen, T. K. , et al. (2017). Aerosols at the poles: An AeroCom phase II multi‐model evaluation. Atmospheric Chemistry and Physics, 17(19), 12197–12218. 10.5194/acp-17-12197-2017

[jgrd57978-bib-0066] Schmale, J. , Baccarini, A. , Thurnherr, I. , Henning, S. , Efraim, A. , Regayre, L. , et al. (2019). Overview of the Antarctic Circumnavigation Expedition: Study of Preindustrial‐like Aerosols and their Climate Effects (ACE‐SPACE). Bulletin of the American Meteorological Society, 100(11), 2260–2283. 10.1175/BAMS-D-18-0187.1

[jgrd57978-bib-0067] Schmale, J. , Zieger, P. , & Ekman, A. M. L. (2021). Aerosols in current and future Arctic climate. Nature Climate Change, 11(2), 95–105. 10.1038/s41558-020-00969-5

[jgrd57978-bib-0068] Schneider, J. , Höhler, K. , Heikkilä, P. , Keskinen, J. , Bertozzi, B. , Bogert, P. , et al. (2021). The seasonal cycle of ice‐nucleating particles linked to the abundance of biogenic aerosol in boreal forests. Atmospheric Chemistry and Physics, 21(5), 3899–3918. 10.5194/acp-21-3899-2021

[jgrd57978-bib-0069] Serreze, M. C. , & Barry, R. G. (2011). Processes and impacts of Arctic amplification: A research synthesis. Global and Planetary Change, 77(1–2), 85–96. 10.1016/j.gloplacha.2011.03.004

[jgrd57978-bib-0070] Shingler, T. , Dey, S. , Sorooshian, A. , Brechtel, F. J. , Wang, Z. , Metcalf, A. , et al. (2012). Characterisation and airborne deployment of a new counterflow virtual impactor inlet. Atmospheric Measurement Techniques, 5(6), 1259–1269. 10.5194/amt-5-1259-2012

[jgrd57978-bib-0071] Shupe, M. D. , Kollias, P. , Persson, P. O. G. , & McFarquhar, G. M. (2008). Vertical motions in Arctic mixed‐phase stratiform clouds. Journal of the Atmospheric Sciences, 65(4), 1304–1322. 10.1175/2007JAS2479.1

[jgrd57978-bib-0072] Shupe, M. D. , Matrosov, S. Y. , & Uttal, T. (2006). Arctic mixed‐phase cloud properties derived from surface‐based sensors at SHEBA. Journal of the Atmospheric Sciences, 63(2), 697–711. 10.1175/JAS3659.1

[jgrd57978-bib-0073] Shupe, M. D. , Persson, P. O. G. , Brooks, I. M. , Tjernström, M. , Sedlar, J. , Mauritsen, T. , et al. (2013). Cloud and boundary layer interactions over the Arctic sea ice in late summer. Atmospheric Chemistry and Physics, 13(18), 9379–9399. 10.5194/acp-13-9379-2013

[jgrd57978-bib-0074] Siegel, K. , Karlsson, L. , Zieger, P. , Baccarini, A. , Schmale, J. , Lawler, M. , et al. (2021). Insights into the molecular composition of semi‐volatile aerosols in the summertime central Arctic ocean using FIGAERO‐CIMS. Environmental Sciences: Atmospheres, 1(4), 161–175. 10.1039/D0EA00023J PMC8262249 34278305

[jgrd57978-bib-0075] Solomon, A. , Shupe, M. D. , Persson, P. O. G. , & Morrison, H. (2011). Moisture and dynamical interactions maintaining decoupled Arctic mixed‐phase stratocumulus in the presence of a humidity inversion. Atmospheric Chemistry and Physics, 11(19), 10127–10148. 10.5194/acp-11-10127-2011

[jgrd57978-bib-0076] Sotiropoulou, G. , Sedlar, J. , Forbes, R. , & Tjernström, M. (2016). Summer Arctic clouds in the ECMWF forecast model: An evaluation of cloud parametrization schemes. Quarterly Journal of the Royal Meteorological Society, 142(694), 387–400. 10.1002/qj.2658

[jgrd57978-bib-0077] Sotiropoulou, G. , Sullivan, S. , Savre, J. , Lloyd, G. , Lachlan‐Cope, T. , Ekman, A. M. , & Nenes, A. (2020). The impact of secondary ice production on Arctic stratocumulus. Atmospheric Chemistry and Physics, 20(3), 1301–1316. 10.5194/acp-20-1301-2020

[jgrd57978-bib-0078] Spreen, G. , Kaleschke, L. , & Heygster, G. (2008). Sea ice remote sensing using AMSR‐E 89‐GHz channels. Journal of Geophysical Research, 113(C2), C02S03. 10.1029/2005JC003384

[jgrd57978-bib-0079] Sprenger, M. , & Wernli, H. (2015). The LAGRANTO Lagrangian analysis tool—Version 2.0. Geoscientific Model Development, 8(8), 2569–2586. 10.5194/gmd-8-2569-2015

[jgrd57978-bib-0080] Stevens, R. G. , Loewe, K. , Dearden, C. , Dimitrelos, A. , Possner, A. , Eirund, G. K. , et al. (2018). A model intercomparison of CCN‐limited tenuous clouds in the high Arctic. Atmospheric Chemistry and Physics, 18(15), 11041–11071. 10.5194/acp-18-11041-2018

[jgrd57978-bib-0081] Stohl, A. (2006). Characteristics of atmospheric transport into the Arctic troposphere. Journal of Geophysical Research, 111(D11), D11306. 10.1029/2005JD006888

[jgrd57978-bib-0082] Thomas, M. A. , Devasthale, A. , Tjernström, M. , & Ekman, A. M. L. (2019). The relation between aerosol vertical distribution and temperature inversions in the Arctic in winter and spring. Geophysical Research Letters, 46(5), 2836–2845. 10.1029/2018GL081624

[jgrd57978-bib-0083] Tjernström, M. , Leck, C. , Birch, C. E. , Bottenheim, J. W. , Brooks, B. J. , Brooks, I. M. , et al. (2014). The Arctic Summer Cloud Ocean Study (ASCOS): Overview and experimental design. Atmospheric Chemistry and Physics, 14(6), 2823–2869. 10.5194/acp-14-2823-2014

[jgrd57978-bib-0084] Tjernström, M. , Svensson, G. , Magnusson, L. , Brooks, I. M. , Prytherch, J. , Vüllers, J. , & Young, G. (2021). Central Arctic weather forecasting: Confronting the ECMWF IFS with observations from the Arctic Ocean 2018 expedition. Quarterly Journal of the Royal Meteorological Society, 147(735), 1278–1299. 10.1002/qj.3971

[jgrd57978-bib-0085] Tobo, Y. , Adachi, K. , DeMott, P. J. , Hill, T. C. J. , Hamilton, D. S. , Mahowald, N. M. , et al. (2019). Glacially sourced dust as a potentially significant source of ice nucleating particles. Nature Geoscience, 12(4), 253–258. 10.1038/s41561-019-0314-x

[jgrd57978-bib-0086] Twohy, C. , Strapp, J. , & Wendisch, M. (2003). Performance of a counterflow virtual impactor in the NASA Icing Research Tunnel. Journal of Atmospheric and Oceanic Technology, 20(6), 781–790. 10.1175/1520-0426(2003)020<0781:POACVI>2.0.CO;2

[jgrd57978-bib-0087] Twohy, C. H. , DeMott, P. J. , Russell, L. M. , Toohey, D. W. , Rainwater, B. , Geiss, R. , et al. (2021). Cloud‐nucleating particles over the southern ocean in a changing climate. Earth’s Future, 9(3), e2020EF001673. 10.1029/2020EF001673

[jgrd57978-bib-0088] Twomey, S. (1977). The influence of pollution on the shortwave albedo of clouds. Journal of the Atmospheric Sciences, 34(7), 1149–1152. 10.1175/1520-0469(1977)034<1149:TIOPOT>2.0.CO;2

[jgrd57978-bib-0089] Uttal, T. , Curry, J. A. , McPhee, M. G. , Perovich, D. K. , Moritz, R. E. , Maslanik, J. A. , et al. (2002). Surface heat budget of the Arctic Ocean. Bulletin of the American Meteorological Society, 83(2), 255–276. 10.1175/1520-0477(2002)083<0255:shbota>2.3.co;2

[jgrd57978-bib-0090] Vüllers, J. , Achtert, P. , Brooks, I. M. , Tjernström, M. , Prytherch, J. , Burzik, A. , & Neely, R., III . (2021). Meteorological and cloud conditions during the Arctic Ocean 2018 expedition. Atmospheric Chemistry and Physics, 21(1), 289–314. 10.5194/acp-21-289-2021

[jgrd57978-bib-0091] Wendisch, M. , Macke, A. , Ehrlich, A. , Lüpkes, C. , Mech, M. , Chechin, D. , et al. (2019). The Arctic cloud puzzle: Using ACLOUD/PASCAL multiplatform observations to unravel the role of clouds and aerosol particles in Arctic amplification. Bulletin of the American Meteorological Society, 100(5), 841–871. 10.1175/BAMS-D-18-0072.1

[jgrd57978-bib-0092] Whaley, C. H. , Mahmood, R. , von Salzen, K. , Winter, B. , Eckhardt, S. , Arnold, S. , et al. (2022). Model evaluation of short‐lived climate forcers for the Arctic Monitoring and Assessment Programme: A multi‐species, multi‐model study. Atmospheric Chemistry and Physics, 22, 5775–5828. 10.5194/acp-22-5775-2022

[jgrd57978-bib-0093] Wiedensohler, A. , Birmili, W. , Putaud, J. , & Orgen, J. (2014). Recommendations for aerosol sampling (pp. 45–59). Wiley Online Library. 10.1002/9781118682555.ch3

[jgrd57978-bib-0094] Willis, M. D. , Leaitch, W. R. , & Abbatt, J. P. (2018). Processes controlling the composition and abundance of Arctic aerosol. Reviews of Geophysics, 56(4), 621–671. 10.1029/2018RG000602

[jgrd57978-bib-0095] Wilson, T. W. , Ladino, L. A. , Alpert, P. A. , Breckels, M. N. , Brooks, I. M. , Browse, J. , et al. (2015). A marine biogenic source of atmospheric ice‐nucleating particles. Nature, 525(7568), 234–238. 10.1038/nature14986 26354482

[jgrd57978-bib-0096] WMO/GAW . (2016). WMO/GAW aerosol measurement procedures, guidelines and recommendations (2nd ed.). Report No. 227. World Meteorological Organization.

[jgrd57978-bib-0097] Yang, X. , Pyle, J. A. , & Cox, R. A. (2008). Sea salt aerosol production and bromine release: Role of snow on sea ice. Geophysical Research Letters, 35(16), L16815. 10.1029/2008GL034536

[jgrd57978-bib-0098] Young, G. , Vüllers, J. , Achtert, P. , Field, P. , Day, J. , Connor, E. O. , et al. (2021). Evaluating Arctic meteorology modelled with the unified model and integrated forecasting system. Atmospheric Chemistry and Physics Discussions, 2021, 1–54. 10.5194/acp-2021-662

[jgrd57978-bib-0099] Baumgardner, D. , Subramanian, R. , Twohy, C. , Stith, J. , & Kok, G. (2008). Scavenging of black carbon by ice crystals over the northern Pacific. Geophysical Research Letters, 35(22), L22815. 10.1029/2008GL035764

[jgrd57978-bib-0100] Field, P. R. , Lawson, R. P. , Brown, P. R. A. , Lloyd, G. , Westbrook, C. , Moisseev, D. , et al. (2016). Chapter 7. Secondary Ice Production—Current state of the science and recommendations for the future. Meteorological Monographs, 58, 7–20. 10.1175/AMSMONOGRAPHS-D-16-0014.1

[jgrd57978-bib-0101] Hänel, G. (1987). Role of aerosol properties during the condensational stage of cloud: A reinvestigation of numerics and microphysics. Beiträge zur Physik der Atmosphäre, 60(3), 321–339.

[jgrd57978-bib-0102] Partridge, D. G. , Vrugt, J. A. , Tunved, P. , Ekman, A. M. L. , Gorea, D. , & Sorooshian, A. (2011). Inverse modeling of cloud‐aerosol interactions—Part 1: Detailed response surface analysis. Atmospheric Chemistry and Physics, 11(14), 7269–7287. 10.5194/acp-11-7269-2011

[jgrd57978-bib-0103] Roelofs, G. (1992). On the drop and aerosol size dependence of aqueous sulfate formation in a continental cumulus cloud. Atmospheric Environment Part A. General Topics, 26(13), 2309–2321. 10.1016/0960-1686(92)90362-O

[jgrd57978-bib-0104] Roelofs, G.‐J. , & Jongen, S. (2004). A model study of the influence of aerosol size and chemical properties on precipitation formation in warm clouds. Journal of Geophysical Research, 109(D22). 10.1029/2004JD004779

[jgrd57978-bib-0105] Seinfeld, J. H. , & Pandis, S. N. (2016). Atmospheric chemistry and physics: From air pollution to climate change (3rd ed.). John Wiley & Sons, Incorporated.

[jgrd57978-bib-0106] Sumlin, B. J. , Heinson, W. R. , & Chakrabarty, R. K. (2018). Retrieving the aerosol complex refractive index using PyMieScatt: A Mie computational package with visualization capabilities. Journal of Quantitative Spectroscopy and Radiative Transfer, 205, 127–134. 10.1016/j.jqsrt.2017.10.012

[jgrd57978-bib-0107] von der Weiden, S.‐L. , Drewnick, F. , & Borrmann, S. (2009). Particle Loss Calculator—A new software tool for the assessment of the performance of aerosol inlet systems. Atmospheric Measurement Techniques, 2(2), 479–494. 10.5194/amt-2-479-2009

[jgrd57978-bib-0108] Wiedensohler, A. , Birmili, W. , Nowak, A. , Sonntag, A. , Weinhold, K. , Merkel, M. , et al. (2012). Mobility particle size spectrometers: Harmonization of technical standards and data structure to facilitate high quality long‐term observations of atmospheric particle number size distributions. Atmospheric Measurement Techniques, 5(3), 657–685. 10.5194/amt-5-657-2012

[jgrd57978-bib-0109] Zieger, P. , Väisänen, O. , Corbin, J. C. , Partridge, D. G. , Bastelberger, S. , Mousavi‐Fard, M. , et al. (2017). Revising the hygroscopicity of inorganic sea salt particles. Nature Communications, 8(May), 15883. 10.1038/ncomms15883 PMC5500848 28671188

