# Supplementary material for: Physical and Chemical Properties of Cloud Droplet Residuals and Aerosol Particles During the Arctic Ocean 2018 Expedition
Source: J Geophys Res Atmos. 2022 Jun 2;127(11):e2021JD036383. doi: 10.1029/2021JD036383 (PMC9285477; doi:10.1029/2021JD036383)
Supplement: Supplementary file 1 — Supporting Information S1 [file JGRD-127-0-s001.pdf]

Supporting Information for "Physical and chemical properties of cloud droplet residuals and aerosol particles during the Arctic Ocean 2018 expedition"

Linn Karlsson[1,2], Andrea Baccarini[3,4], Patrick Duplessis[5], Darrel Baumgardner[6], Ian M. Brooks[7], Rachel Y.-W. Chang[5], Lubna Dada[3,4], Kaspar R. Dällenbach[4], Liine Heikkinen[1,2], Radovan Krejci[1,2], W. Richard Leaitch[8], Caroline Leck[2,9], Daniel G. Partridge[10], Matthew E. Salter[1,2], Heini Wernli[11], Michael J. Wheeler[12], Julia Schmale[3], and Paul Zieger[1,2]

[1]Department of Environmental Science, Stockholm University, Sweden

[2]Bolin Centre for Climate Research, Stockholm University, Sweden

[3]Extreme Environments Research Laboratory, École Polytechnique fédérale de Lausanne, Switzerland

[4]Laboratory of Atmospheric Chemistry, Paul Scherrer Institute, Switzerland

[5]Department of Physics and Atmospheric Science, Dalhousie University, Canada

[6]Droplet Measurement Technologies, LLC, USA

[7]Institute for Climate and Atmospheric Science, School of Earth and Environment, University of Leeds, United Kingdom

[8]Climate Research Division, Environment and Climate Change Canada, Canada

[9]Department of Meteorology, Stockholm University, Sweden

[10]College of Engineering, Mathematics and Physical Sciences, University of Exeter, United Kingdom

[11]Department of Environmental Systems Science, ETH Zürich, Switzerland

[12]Air Quality Research Division, Environment and Climate Change Canada, Canada

---

The supplementary information is structured as follows:

Section 1 describes the back-trajectory sensitivity analysis. This section also includes other supplement figures related to trajectories.

Section 2 is about the GCVI sampling efficiency, which describes how different proxies of the cloud particle number concentration can be used to estimate the GCVI sampling efficiency, which method was chosen, and why.

Section 3 includes all supplement figures and tables that relate to the aerosol mass spectrometer (AMS) data, including more details on the limits of detection.

Section 4 includes additional information on the overall data treatment.

Section 5 describes the cloud parcel modeling results.

Section 6 includes all supplement figures and tables that did not fit into any of the above categories.

1. Trajectory sensitivity analysis

The Arctic boundary layer is notoriously difficult to model (Birch et al., 2012; Sotiropoulou et al., 2016; Graham et al., 2019; Tjernström et al., 2021; Young et al., 2021). This could have effects on the analysis, mainly Fig. 5j in the main manuscript. Fig. S1 compares the fraction over ocean/land/ice/above boundary layer for different boundary layer heights (BLH) – the model output BLH, the model output BLH$\pm$15hPa, and a constant BLH (the mean model BLH for all the trajectory data points, around 965 hPa). The overall patterns are very similar, so the results are not sensitive to these variations in the BLH.

Finally, the "origin" of an air parcel is not necessarily well-defined. We elected to use 5-day back trajectories to balance aerosol lifetime with trajectory uncertainties (increasing

with the number of days). Figure S2 compares the ice/ocean/land/above boundary layer fractions 3-, 5-, 7-, and 10-day trajectories. Shorter trajectories result in a slightly higher fraction of time spent over ice, the longest trajectories spend longer above the boundary layer and slightly more time over land, but overall the patterns are similar. Figure S3 tells a similar story for the trajectories related to cloud events and cloud residual number mean diameter bins. The ocean influence relative to ice increases with cloud residual NMD, and this trend is slightly stronger for longer trajectories.

2. GCVI sampling efficiency

The GCVI does not sample the cloud particle distributions with an efficiency of unity (Shingler et al., 2012; Karlsson et al., 2021). In order to retrieve concentrations of cloud residuals at representative ambient concentrations, we therefore need to determine the effective sampling efficiency. With our set-up, this can be achieved by comparing the cloud residual number concentrations to:

a) the ambient cloud particle concentration, determined by integrating the FSSP cloud particle size distribution,

b) the difference between the particle concentrations measured by the whole-air inlet and the interstitial inlet, and/or

c) the whole-air particle size distribution for warm clouds, assuming larger particles activate first (i.e. comparing the accumulation mode concentration of cloud residuals to that of the whole air particles).

a) Cloud particle concentration comparison: Cloud particle number size distributions were measured by a forward scattering spectrometer probe (FSSP; Particle Metrics Inc., USA, Model FSSP-100), measuring particles from 0.5 to 47 um diameter. Unfortunately, we were not able to use the measured cloud particle size distributions from the FSSP to infer the GCVI sampling efficiency. Figure S6b shows a density scatter plot of the liquid water content (LWC) as measured by a PVM versus the LWC calculated from the FSSP size distributions by assuming spherical droplets with the density of water. The calculated LWC is only about 30 % of the measured LWC, which suggests that the cloud particle concentrations measured by the FSSP were too low. Figure S6c shows a density scatter plot of the visibility measured by the sensor next to the GCVI versus

visibility calculated from the FSSP size distributions assuming spherical particles and using the Koschmeider formula (Seinfeld & Pandis, 2016) and the `PyMieScatt` Python package (Sumlin et al., 2018). The calculated visibility is, on average, several times higher than the measured one, which, again, suggests that the FSSP was undercounting cloud particles. The FSSP laser failed on Sep 6, so it is possible that there were technical issues leading up to the failure.

b) Total minus interstitial concentration comparison: The total minus interstitial particle concentration should, in theory, be on the same order of magnitude as the total cloud particle concentration, since we are subtracting the non-activated (interstitial) particles from the total particles which include activated particles (i.e. cloud particles). Therefore, comparing this difference to the cloud residual concentrations could give an indication of the transmission efficiency of the GCVI inlet.

Figure S7a shows a density scatter plot of the difference between total and interstitial particle concentrations measured by the CPCs versus the concentration of cloud residuals. The orthogonal distance linear regression resulted in a slope of about 6 (i.e. a sampling efficiency of about 17 %); however, the correlation between the two concentrations is quite weak ($\rho = 0.3$) and there are data points around the 1:1 line which would indicate that the cloud residual number concentration is too high, or that the total minus interstitial concentration is too low or does not accurately represent the cloud particle concentration.

CPC intercomparisons performed weekly during the expedition showed that the interstitial CPC on average measured 10% higher concentrations than the whole-air CPC (within standard measurement uncertainty: Wiedensohler et al., 2012), which can be part of the explanation why the total minus interstitial concentration sometimes appears too low.

c) Accumulation mode concentration comparison: The third method compares accumulation mode particle number concentrations of the cloud residuals to that of the whole air particles. This comparison can only be done when the clouds are mostly liquid, since it is based on the assumption of liquid droplet activation in which accumulation mode particles activate first. The comparison was made for clouds at temperatures above -2°C for a range of different boundaries, D_{cut}, between the Aitken and accumulation mode. The number size distributions were integrated between D_{cut} and 921 nm (i.e. the last size bin) to get the accumulation mode concentrations for the comparison. The temperature boundary was not put at 0°C because that would only have included the cloud event during the first MIZ station which might not be representative for all the data. Ice nucleation was not observed above -5°C during AO18 or other Arctic studies (Porter et al., 2021, and references therein), so the boundary of -2°C should be sufficient to eliminate the presence of the vast majority of ice particles.

To determine the ideal D_{cut}, the slope was chosen for the case with the highest coefficient of determination of $R^2 = 0.92$ (Fig. S7b and c), which resulted in a sampling efficiency of about 6 % (slope of 16.93) at a correlation coefficient of $\rho = 0.56$. This is likely the most trustworthy of the estimated GCVI sampling efficiencies, due to the weak correlation in the total minus interstitial method and the fact that the FSSP cloud particle size distribution is somewhat questionable and could not be used. Karlsson et al. (2021) evaluated these approaches for a similar GCVI, and the accumulation mode comparison in their study agreed very well with the sampling efficiency as determined by using the ambient cloud particle size distributions. As such, we have corrected all cloud residual size distributions by a factor of 17 (rounded value of 16.93).

The factor 17 is significantly higher than the average factor of around 2.2 that was observed in Karlsson et al. (2021). Part of this difference can be explained by the fact that the GCVI transmission efficiency is size dependent (Shingler et al., 2012) and the fact that the cloud droplet size distributions differ between AO18 and Zeppelin Observatory. Because the FSSP data were questionable and the concentrations appeared too low, we did not use them to estimate the overall transmission efficiency. If we nevertheless assume that the shape of the cloud droplet size distribution measured by the FSSP is reasonably accurate, we can compare the theoretical fraction of sampled droplets between AO18 and Zeppelin Observatory based on the different cut sizes of the GCVI inlets used and on the extrapolated size-dependent transmission efficiency from Shingler et al. (2012) used in Karlsson et al. (2021). Figure S8 below shows that we theoretically could sample about 14% of the cloud droplets during AO18, as opposed to 46% at Zeppelin Observatory (from Karlsson et al., 2021). This is still around a factor of 2 off from the 6% obtained by the accumulation mode comparison and we cannot explain the last part of the difference except to emphasize that there are many uncertainties involved in the comparison presented here.

We are aware that using a constant correction factor is not ideal, and the concentrations obtained this way should be considered approximate. For looking at average cloud residual size distributions, a constant factor should be sufficient, but for individual size distributions the concentrations could be either under- or overestimated. However, the correction factor 17 leads to cloud residual number concentrations that are in a similar range as CCN concentrations reported from previous expeditions (cf. Bigg & Leck, 2001; Mauritsen et al., 2011; Leck & Svensson, 2015).

3. AMS-related figures and tables

Detection limits (LOD) of the AMS:

The limit of detection (LOD) for each compound was calculated from the mean plus one standard deviation of the signal during HEPA filter background measurements. The mean background and standard deviations for each compound at 1 min time resolution are listed in Tab. S1 below.

Detection limits for the relevant averaging periods were calculated according to Eq. 1.

$$\mathrm{LOD}_{n\,\mathrm{min}} = \mathrm{BG} + \frac{\mathrm{SD}}{\sqrt{n}} \tag{1}$$

The tables that follow show the measured concentrations for the AMS data from the figures in the main manuscript. Values where the signal is weaker than the LOD are marked in red.

Figure S12 shows a modified version of Fig. 5 in the main manuscript, in which data points with values that lie below the LOD have been marked in gray (all LODs were calculated according to Eq. 1 based on the exact number of data points included in the average). Note that the top row in Fig. S12 shows that the correlations discussed in the main manuscript still remain (although weaker for sulfate and stronger for organics) even if only above-LOD data are considered.

4. Additional information on data treatment

4.1. Loss correction

All DMPS and SMPS particle number size distribution data were corrected for diffusion, impaction and sedimentation losses using the *Particle Loss Calculator* (von der Weiden et al., 2009), assuming spherical particles with a density of $1.5\,\mathrm{g\,cm^{-3}}$ which accounts for the presence of organic substances with low density (e.g. $1.3\ \mathrm{g\,cm^{-3}}$, Siegel et al., 2021) and inorganic salts such as ammonium sulfate ($1.77\ \mathrm{g\ cm^{-3}}$) or sea salt (e.g. $2.0\ \mathrm{g\,cm^{-3}}$, Zieger et al., 2017). Overall, the SMPS and both DMPS systems agreed well in terms of the modal diameters of the aerosol size distributions, but the SMPS often showed higher concentrations than both DMPSs in both aerosol size modes (but particularly the Aitken mode, see Fig. S14) which could be due to the different inlet lines and/or remaining differences in transmission efficiencies (e.g. within the instruments, since only line losses were accounted for). This limits us to studying the total minus interstitial size distributions as averages over long periods, when the SMPS can be compared to itself since it was alternating between inlets every hour (see Fig. S13).

4.2. Pollution flag

The lab containers and inlets were set up such that they were upstream of the ship exhaust as long as *Oden* faced into the wind. The bow of the ship was kept facing into the wind as much as possible to avoid pollution from the ship stack contaminating the measurements. In addition, activities on the front deck were restricted and all smaller exhausts (e.g. ventilation from containers) were piped to the back of the ship.

A pollution flag was developed based on 1 s resolution number concentration data from the MCPC behind the whole-air inlet. When the MCPC concentration range in a 30 s

period exceeded the mean absolute concentration over a 6 h period centered on the same point, and the range exceeded 50 cm^{-3}, then the data point was flagged as contaminated. The minimum requirement on the range was added to limit the flagging of uncontaminated data points when the total number concentrations were low. In addition to this, all points where the concentration exceeded 10^4 cm^{-3} were flagged (this does not eliminate new particle formation events as concentrations above the MCPC cut size remained below this threshold during the events defined in Baccarini et al. (2020)), as well as any periods noted in the logbook as potentially influenced by pollution. Finally, the number concentration time series was visually inspected to flag any remaining outliers and suspicious data points.

Lower-resolution data were cleaned based on the 1 s flag, such that if suspected contamination occurred at any point during the measurement interval, it was flagged as contaminated.

4.3. Definition of cloudy periods

As previously stated, the GCVI was run in manual mode during the AO18 expedition, so whether the air we sampled qualified as "cloudy" or not needed to be decided in post-processing. To do this, we used a combination of the visibility data recorded by the GCVI and the LWC measured by the PVM. To achieve an optimal data coverage, we chose a maximum visibility of 2 km in combination with an LWC of more than 0.01 $g\,m^{-3}$ to be considered as cloudy conditions. Events where such conditions persisted for at least 27 min (i.e. a minimum of 3 DMPS scans) were classified as cloud events and are included in the data presented in this paper. All in all, there were 25 cloud events with a total of 327 measured cloud residual size distributions, or 48.8 hours of in-cloud measurements. Note that this does not correspond to the total cloud occurrence during the expedition, since

the GCVI was not continuously sampling. During the included cloud events, the average LWC ranged from approximately 0.01 to 0.2 $\mathrm{g\,m^{-3}}$, while the effective cloud particle radius (measured by the PVM) ranged between 7.0 and 9.2 μm (25th and 75th percentiles). This is in good agreement with the FSSP data which had an effective radius between 7.7 and 12.2 (main droplet number mode around 8 μm diameter). The CVI cut-off only allows us to sample a subset of the cloud particle distribution and we can only assume that there is no partitioning of physical and chemical cloud residual properties between the smaller and larger cloud particles. This assumption, however, would need more sophisticated experimental work to be proved or disproved. It is also important to note that cloud residuals do not necessarily directly correspond to CCN or INP – the particles can be modified by other atmospheric processes like scavenging (e.g. Baumgardner et al., 2008) or secondary ice processes (e.g. Field et al., 2016).

5. Cloud parcel modelling

A pseudo-adiabatic cloud parcel model (G.-J. Roelofs & Jongen, 2004) further developed to allow for adiabatic ascent of the air parcel (Partridge et al., 2011, 2012), and a description of surface-active organics (Lowe et al., 2019) was used to estimate the smallest activation (dry) diameters and fraction of aerosol particles that activate into cloud droplets using different chemical compositions and updraft velocities. Aerosol activation and condensation/evaporation of water are calculated according to the Köhler equation and parameterized according to Hänel (1987); G. Roelofs (1992). For the simulations performed, the model is initialised with a relative humidity (90%), temperature (270 K), and pressure (980 hPa). The initial dry aerosol size distribution is described using the log-normal distribution fit parameters for the aerosol number size distribution modes present (Table 1 whole air in 32–79 nm size range, see main manuscript), the aerosol chemical composition and updraft velocity. Particles with wet radii larger than the critical radii as predicted by Köhler theory were counted as cloud droplets. In the simulations shown here, both Aitken and Accumulation mode had the same composition. We performed three different modelling scenarios under a range of updraft velocities which spans the approx. range of the observed distribution in Arctic stratus (Shupe et al., 2013) (from $0.1\,\mathrm{ms}^{-1}$ to $1\,\mathrm{ms}^{-1}$):

- Assuming the aerosol particles comprised 70% organics (Org) and 30% ammonium sulfate (AS). The organics were assumed to be soluble and the particles well mixed. These simulations are therefore referred to as the bulk Köhler, BK (Org+AS) simulations.

- Assuming all the particles comprised sulfuric acid (SA) to probe the impact of extremely hygroscopic Aitken mode on the minimum activation diameter size. These simulations are referred to as the BK (SA) simulations.

- Assuming the same composition as for the BK (Org + AS), but only 50 % of the organics were treated as soluble. The insoluble organics were instead treated as surface active forming a minimum 0.2 nm thick layer onto the particle surface. This organic surface layer had a surface tension set to 40 mNm^{-1}. For more information on how the surface-active organics are described we direct the readers to Lowe et al. (2019). These simulations are referred to as the compressed film, CF (Org+AS) simulations.

Figure S15 shows the vertical evolution of the parcel model size bins for the BK (SA) case under 0.5 ms^{-1} updraft. At maximum supersaturation the activated size bins have clearly diverged from the unactivated bins.

6. Further figures and tables

- Table S5 presents the list of instruments, measured parameters and their temporal resolution.

- Figure S17 shows an overview of cloud residual size distributions and corresponding total particle size distributions.

- Figure S18 shows an overview of the cloud residual number mean diameter, the cloud residual Aitken mode fraction, the the cloud residual number concentration and the whole-air particle number concentration.

- Figure S19 shows the individual fits from Fig. 4 and Table 1 in the main manuscript.

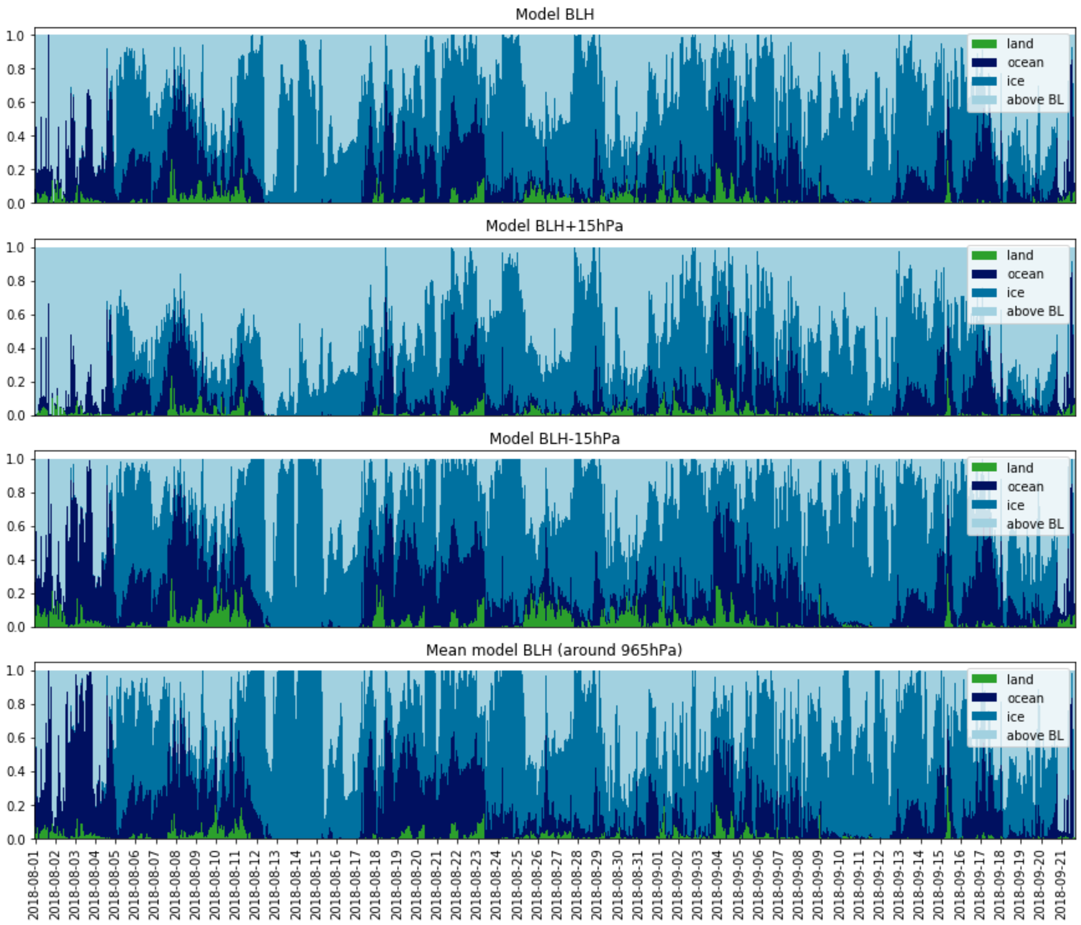

Figure S1. Trajectory time fractions spent over ice, ocean, land, or above the boundary layer, compared for different boundary layer heights (BLH).

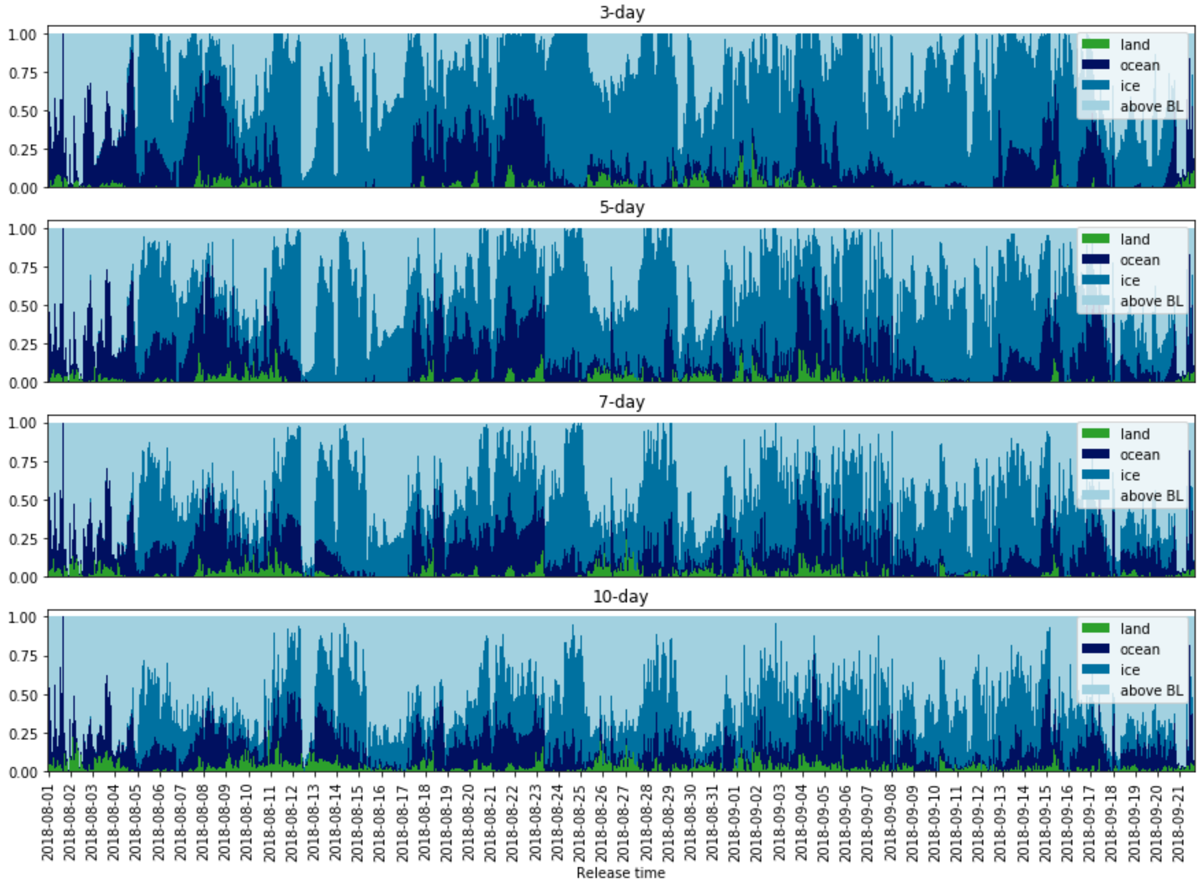

Figure S2. Trajectory time fractions spent over ice, ocean, land, or above the boundary layer, compared for 3-, 5-, 7-, and 10-day back trajectories.

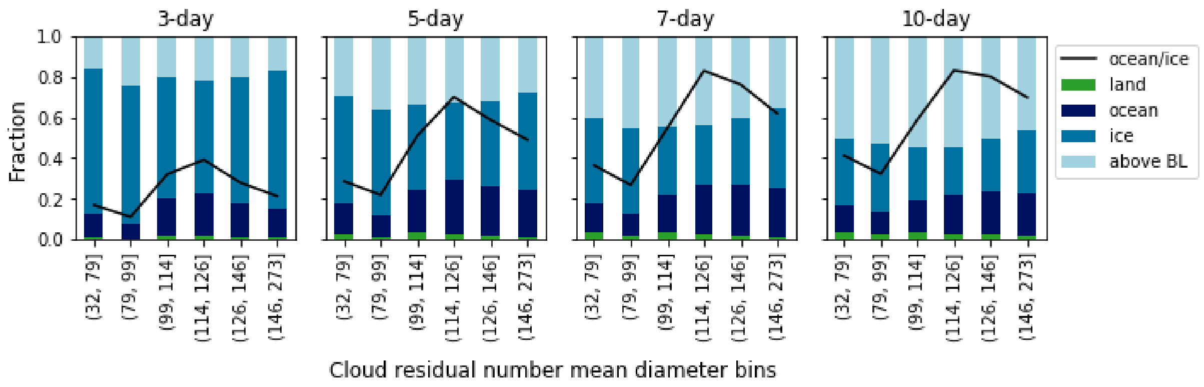

Figure S3. Trajectory time fractions spent over ice, ocean, land, or above the boundary layer, for each cloud residual number mean diameter bin (i.e. cloud events during the ice drift), compared for 3-, 5-, 7-, and 10-day back trajectories. See also Fig. 5j in main manuscript.

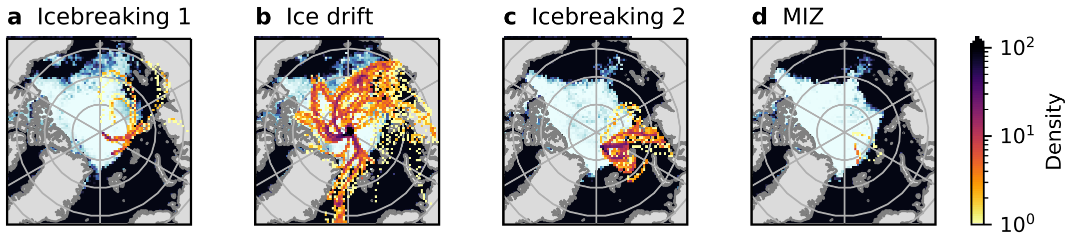

Figure S4. Back trajectories for the cloud residual groups in Fig. 3 in the main manuscript, but here only including points where the trajectories are within the boundary layer ($p > p_{BL}$).

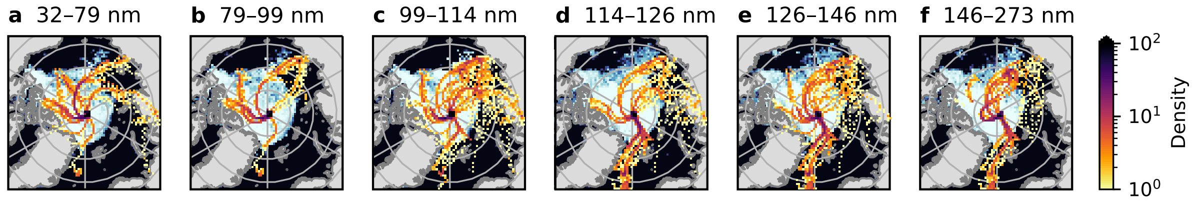

Figure S5. Back trajectories for the cloud residual groups in Fig. 4 in the main manuscript, but here only including points where the trajectories are within the boundary layer ($p > p_{BL}$).

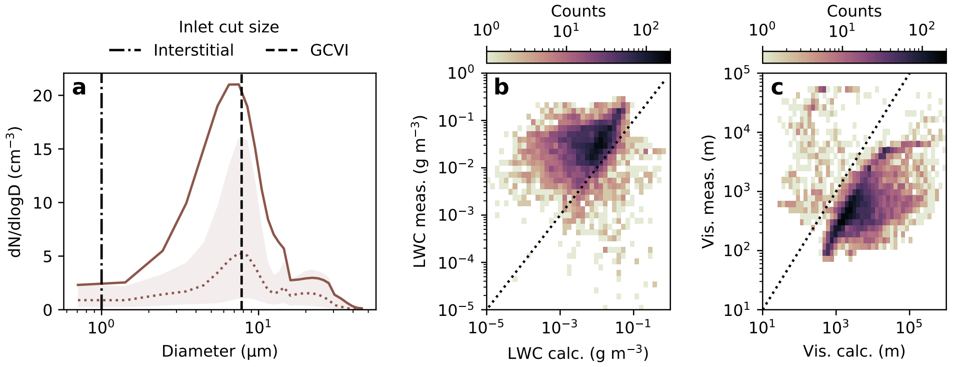

Figure S6. **a** Cloud particle size distribution during cloudy conditions (visibility $< 2\,\mathrm{km}$ and liquid water content (LWC) $> 0.01\ \mathrm{g\,m^{-3}}$), as measured with an FSSP. Solid and dotted lines show mean and median values, respectively, and shaded areas indicate the 25th to 75th percentile ranges. The cut size diameter of the interstitial and GCVI inlets are also indicated in the figure. **b** density scatter plot of LWC measured with a PVM versus LWC calculated from FSSP size distributions (assuming spherical particles with the density of water). **c** density scatter plot of the visibility measured by the sensor next to the GCVI inlet versus the visibility calculated from FSSP size distributions (assuming spherical particles and using the Koschmeider formula (Seinfeld & Pandis, 2016) and the `PyMieScatt` Python package (Sumlin et al., 2018)). In **b** and **c**, the black dashed line is the 1:1 line. Note that **b** and **c** include all data points where both FSSP and PVM/GCVI were measuring, while **a** only includes data that meet the stricter cloud criteria.

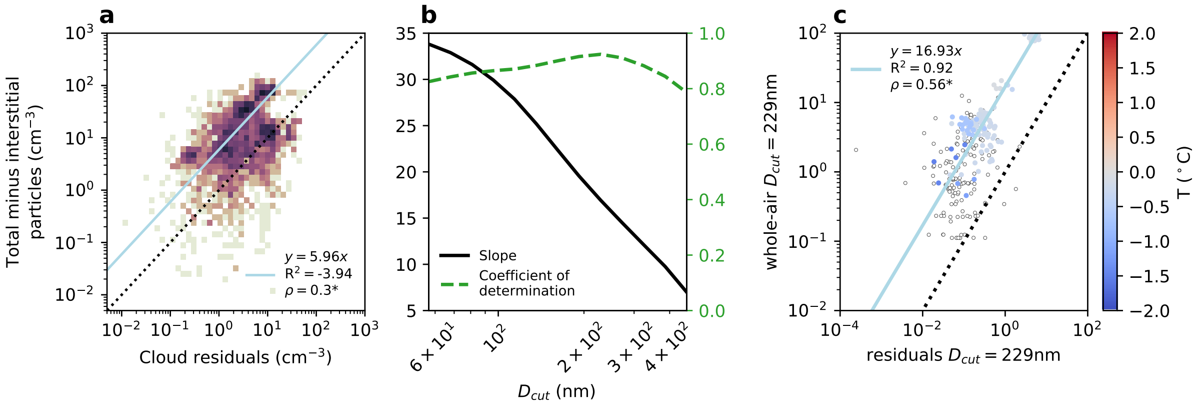

Figure S7. Comparing cloud residuals and cloud proxies. **a** density scatter plot of total-minus-interstitial aerosol particle concentration versus cloud residual concentration, together with an orthogonal distance regression (ODR; blue line), coefficient of determination (R^2), and the Spearman ρ correlation coefficient (asterisk indicates p-value < 0.05). Panels **b** and **c** relate to the comparison of cloud residual and total particle accumulation mode (cloud proxy assuming liquid droplet activation). **b** shows ODR best fit slopes (black line) and corresponding coefficients of determination (dashed green line) for different lower boundaries (D_{cut}) of the accumulation mode. The fits were made for data at temperatures > -2°C. **c** shows a scatterplot of cloud residual and total particle accumulation mode concentrations for the case with the highest coefficient of determination ($\mathrm{R}^2 = 0.92$ at $D_{cut} = 229$ nm) together with the ODR best fit (blue line) and Spearman ρ correlation coefficient. Data for temperatures ≤ -2°C are shown as open gray circles but are not included in the fit (these points make up roughly 60 % of the data). In panels **a** and **c**, the dashed black line indicates the 1:1 line.

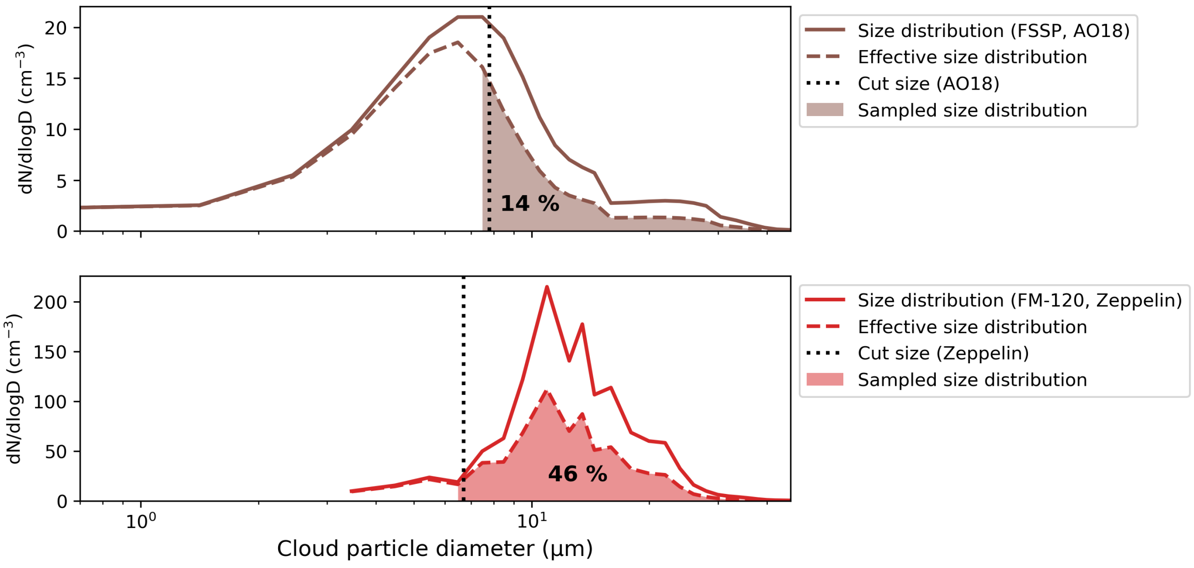

Figure S8. Comparison of theoretically sampled droplets during AO18 and from Zeppelin Observatory (Karlsson et al., 2021). Solid lines show the mean cloud particle size distribution measured by the respective cloud probes. Dashed lines show the effective cloud particle size distribution, i.e. multiplied by the extrapolated Shingler et al. (2012) transmission efficiency. Dotted black lines show the cut sizes of the GCVI systems. Shaded regions show the theoretically sampled particles, and the approximate percentage of the total size distribution is indicated.

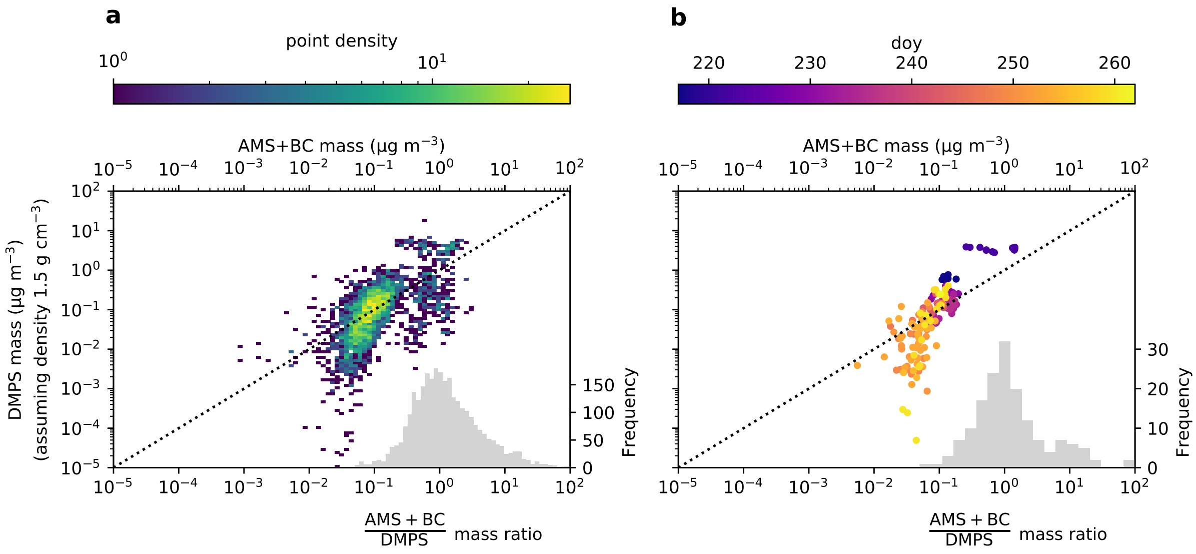

Figure S9. **a** Particle mass calculated from the whole-air DMPS size distributions, by assuming spherical particles with a density of $1.5\,\mathrm{g\,cm^{-3}}$, plotted versus the whole-air mass of the chemical species measured by the AMS plus the equivalent black carbon mass. The colorbar shows the data point density. In the lower right corner, a histogram of the mass ratio ((AMS+BC)/DMPS) is also shown in gray. **b** Same parameters as in a, but plotted only for the cloud events in the main paper (color-coded by day of year instead of point density due to the lower number of data points). In both panels, the dashed black line is the 1:1 line.

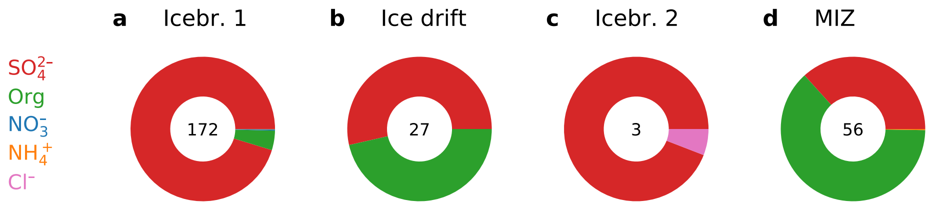

Figure S10. Doughnut plots of mass fractions of whole-air-minus-interstitial (proxy for cloud residuals) AMS aerosol composition. Cf. Fig. 3 in main manuscript. Numbers in the centers of the doughnuts indicate the difference between whole-air and interstitial aerosol mass in ng m^{-3}. Note that the whole-air and interstitial data are not concurrent, which can cause negative values (see first panel).

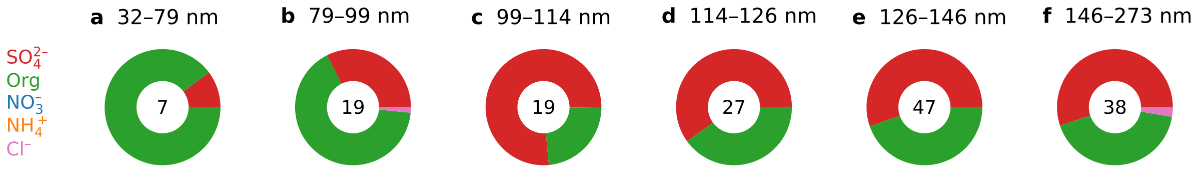

Figure S11. Doughnut plots of mass fractions of whole-air-minus-interstitial (proxy for cloud residuals) AMS aerosol composition. Cf. Fig. 4 in main manuscript. Numbers in the centers of the doughnuts indicate the difference between whole-air and interstitial aerosol mass in ng m^{-3}. Note that the whole-air and interstitial data are not concurrent.

Table S1. Mean background (BG) and standard deviation (SD) of filter measurements for each AMS compound. All values are given in $\mu g\,m^{-3}$. Note that the background for chloride is zero because of the background-subtraction (BG before subtraction was $0.020437\,\mu g\,m^{-3}$).

	Sulfate	Organics	Nitrate	Ammonium	Chloride
BG	0.000521	0.011264	-0.000229	0.000000	
SD	0.007291	0.010546	0.000837	0.006206	

Table S2. Concentration for the AMS compounds sulfate and organics, for the ratios shown in Fig. 2 in the main manuscript (both whole-air and interstitial). Values below the LOD are marked in red, and all LODs were calculated according to Eq. 1 based on the exact number of data points included in the average.

Time	Whole-air		Interstitial		Time	Whole-air		Interstitial	
	SO_4^{2-}	Organics	SO_4^{2-}	Organics		SO_4^{2-}	Organics	SO_4^{2-}	Organics
05 Aug 10H	0.0053	0.11	0.027	0.66	06 Sep 10H	0.0016	0.032	6.6e-05	0.011
10 Aug 20H	0.92	0.46	–	–	06 Sep 11H	5.3e-05	0.034	0.00083	0.004
10 Aug 21H	0.66	0.32	0.39	0.16	08 Sep 13H	–	–	0.0042	0.027
10 Aug 22H	0.35	0.12	–	–	08 Sep 18H	0.0021	0.031	0.0056	0.028
10 Aug 23H	–	–	0.27	0.055	08 Sep 21H	0.0024	0.033	0.004	0.048
18 Aug 16H	0.059	0.08	0.013	0.035	10 Sep 07H	0.00058	0.028	-0.00024	0.039
18 Aug 17H	–	–	–	–	10 Sep 12H	0.0012	0.029	0.0013	0.022
18 Aug 18H	–	–	–	–	10 Sep 16H	0.0033	0.031	0.0019	0.04
18 Aug 19H	–	–	–	–	10 Sep 17H	0.0029	0.051	0.00065	0.044
18 Aug 20H	0.069	0.078	0.019	0.032	10 Sep 18H	0.0041	0.035	0.005	0.022
18 Aug 21H	0.066	0.08	0.027	0.06	10 Sep 19H	5.4e-05	0.046	0.0014	0.049
18 Aug 21H	0.047	0.057	0.016	0.049	10 Sep 20H	0.004	0.04	0.0054	0.043
22 Aug 18H	0.047	0.069	0.0013	0.037	11 Sep 10H	0.028	0.077	0.0065	0.021
22 Aug 19H	0.061	0.091	0.0057	0.041	11 Sep 14H	0.01	0.051	0.004	0.035
22 Aug 20H	0.027	0.051	0.0051	0.033	11 Sep 15H	0.014	0.034	0.0041	0.061
22 Aug 20H	0.026	0.04	–	–	11 Sep 16H	0.0064	0.043	0.0048	0.023
24 Aug 16H	0.056	0.09	0.017	0.077	11 Sep 16H	0.0067	0.04	0.00062	0.034
24 Aug 17H	0.057	0.062	0.017	0.055	11 Sep 17H	0.0061	0.049	0.00071	0.026
24 Aug 18H	0.059	0.075	0.011	0.044	12 Sep 20H	0.00043	0.037	–	–
26 Aug 19H	–	–	0.0007	0.039	16 Sep 11H	0.047	0.077	0.017	0.046
31 Aug 14H	0.026	0.049	0.0072	0.031	16 Sep 12H	0.035	0.064	0.03	0.074
31 Aug 15H	0.024	0.063	0.0059	0.038	16 Sep 19H	–	–	0.026	0.064
31 Aug 16H	0.016	0.046	0.0055	0.033	16 Sep 21H	0.064	0.094	0.0034	0.044
03 Sep 19H	–	–	0.00061	0.025	16 Sep 22H	0.005	0.048	0.0019	0.046
04 Sep 00H	0.0019	0.021	0.0035	0.023	17 Sep 04H	0.0011	0.028	0.0025	0.056
05 Sep 17H	0.017	0.035	0.0051	0.044	17 Sep 07H	0.0072	0.041	0.0013	0.039
06 Sep 04H	0.0081	0.033	0.0055	0.029	17 Sep 08H	0.015	0.062	0.034	0.19
06 Sep 08H	0.0093	0.036	–	–	19 Sep 09H	0.025	0.067	0.0047	0.031
06 Sep 09H	0.0025	0.029	0.00096	0.015	–	–	–	–	–

Table S3. Concentration for the AMS compounds, for the doughnut plots shown in Fig. 3 in the main manuscript (both whole-air and interstitial). Values below the LOD are marked in red, and all LODs were calculated according to Eq. 1 based on the exact number of data points included in the average.

		SO_4^{2-}	Organics	NO_3^-	NH_4^+	Cl^-
interstitial	Icebr. 1	0.2800	0.23	0.012	2e-05	-0.0072
	Ice drift	0.0057	0.035	0.0066	-0.00022	0.00057
	Icebr. 2	0.0120	0.06	-0.0014	-0.00022	0.00067
	MIZ	0.0047	0.031	-0.0038	-0.00042	0.0011
whole-air	Icebr. 1	0.4500	0.24	0.012	0.00013	-0.0062
	Ice drift	0.0210	0.048	0.0061	-0.00013	-0.00018
	Icebr. 2	0.0210	0.055	-0.0016	-5.8e-05	0.0012
	MIZ	0.0250	0.067	-0.0085	0.00014	0.00026

Table S4. Concentration for the AMS compounds, for the doughnut plots shown in Fig. 4 in the main manuscript (both whole-air and interstitial). Values below the LOD are marked in red, and all LODs were calculated according to Eq. 1 based on the exact number of data points included in the average.

		SO_4^{2-}	Organics	NO_3^-	NH_4^+	Cl^-
interstitial	32–79 nm	0.0013	0.029	0.0031	-0.00029	0.00088
	79–99 nm	0.0029	0.027	0.005	-0.00023	0.0003
	99–114 nm	0.0056	0.044	0.0061	-0.00025	0.00086
	114–126 nm	0.0071	0.035	0.0071	-0.00015	0.0011
	126–146 nm	0.0051	0.035	0.008	-0.0002	0.00043
	146–273 nm	0.0140	0.043	0.013	-0.00014	-0.00031
whole-air	32–79 nm	0.0021	0.036	0.003	-0.00014	-0.0001
	79–99 nm	0.0096	0.041	0.003	-0.00013	0.00062
	99–114 nm	0.0210	0.048	0.0062	-0.00014	-3.8e-06
	114–126 nm	0.0240	0.047	0.0071	-4.1e-05	-0.00094
	126–146 nm	0.0320	0.057	0.0073	-0.00018	-0.0018
	146–273 nm	0.0370	0.06	0.011	-0.00016	0.0011

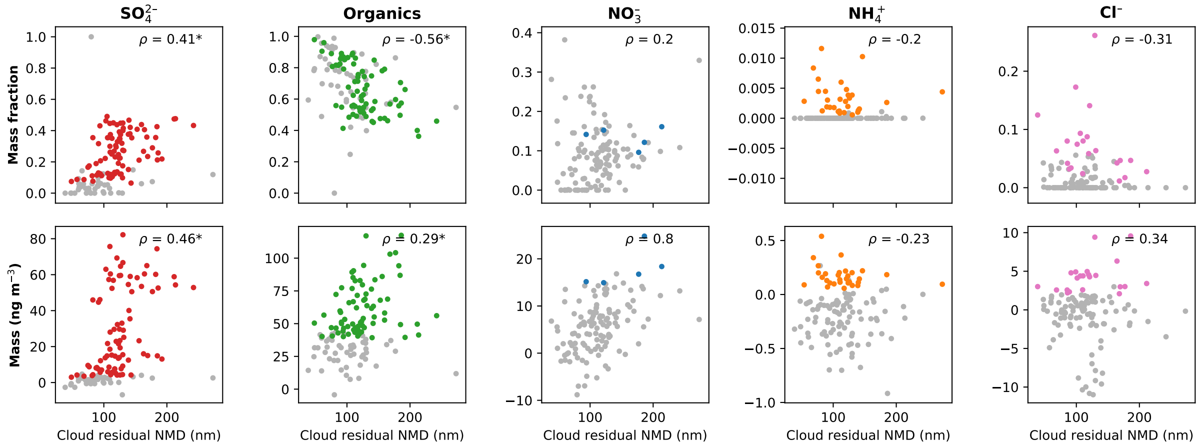

Figure S12. **Top row:** Whole-air mass fractions of sulfate, organics, nitrate, ammonium, and chloride versus cloud residual number mean diameter, together with Spearman ρ correlation coefficients (with an asterisk if the p-value < 0.05) **Bottom row:** Same as top row, but with absolute mass instead of mass fractions on the y-axes. In all panels, data points below the detection limit (LOD) have been colored gray, and only data points above the LOD have been included in the correlation coefficient calculation (cf. Fig. 5 in main manuscript).

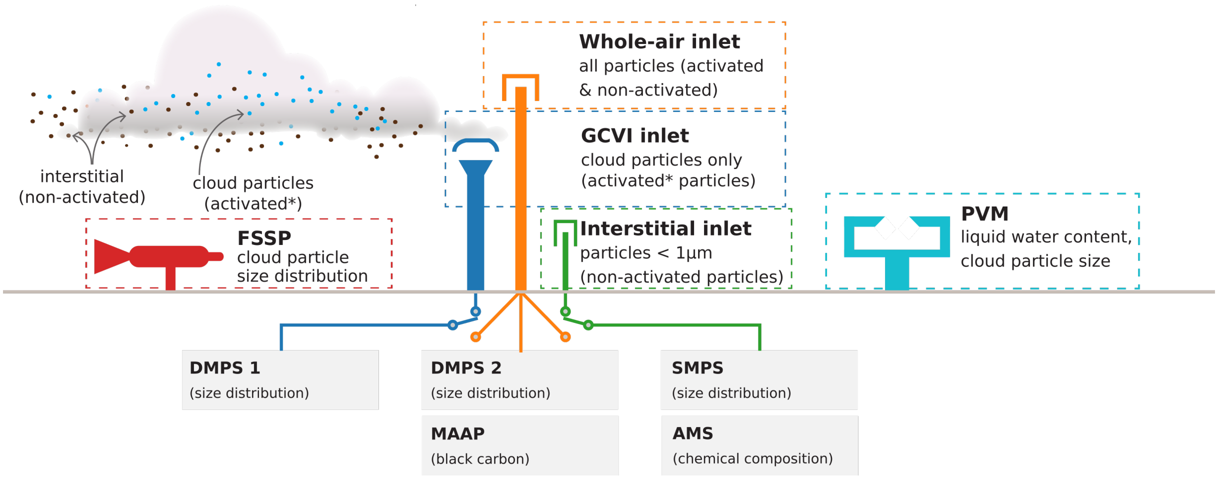

Note that cloud residuals, i.e. particles measured downstream of the GCVI inlet, may not always directly correspond to activated particles.

Figure S13. Diagram of the instrumental set-up. Top half: On the lab container roof. Three inlets (whole-air, GCVI, interstitial) and two cloud probes (forward scattering spectrometer probe, FSSP, and particle volume meter, PVM). Lower half: Inside the lab container(s). Instruments relevant for this study are listed, and the diagram shows which inlet they sampled from. Two valve switches were installed so that some instruments could alternate between the whole-air and GCVI inlets or between the whole-air and interstitial inlets.

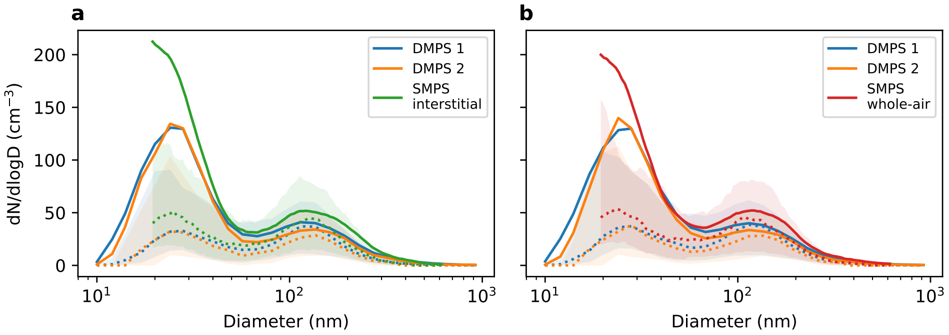

Figure S14. Intercomparison of the two DMPSs and the SMPS during strictly non-cloud periods (min. visibility 10 km, liquid water content less than 0.01 g m^{-3}). **a** odd hours (SMPS on interstitial inlet), **b** even hours (SMPS on whole-air inlet). Solid and dotted lines show mean and median values, respectively, and shaded areas indicate the 25th to 75th percentile ranges.

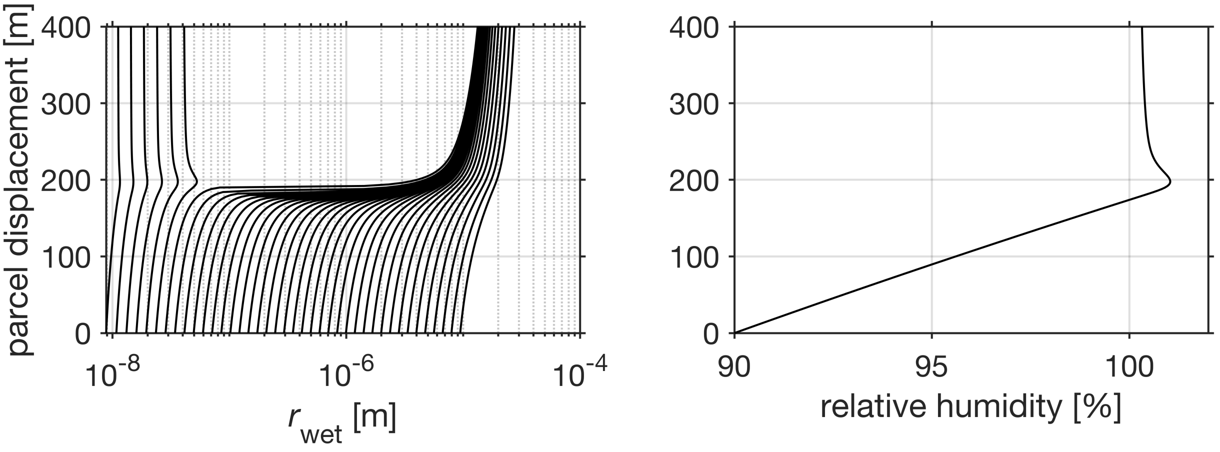

Figure S15. The vertical evolution of the wet particle size bins within the parcel model. The divergence in growth of the activated solution droplets that occurs at maximum supersaturation shows the separation between size bins that remain unactivated, and the growth of the activated cloud droplets particles by condensation during continued ascent. The figure visualizes every 10th size bin (40 bins out of 400) and represents the BK (SA) case with 0.5 m/s updraft.

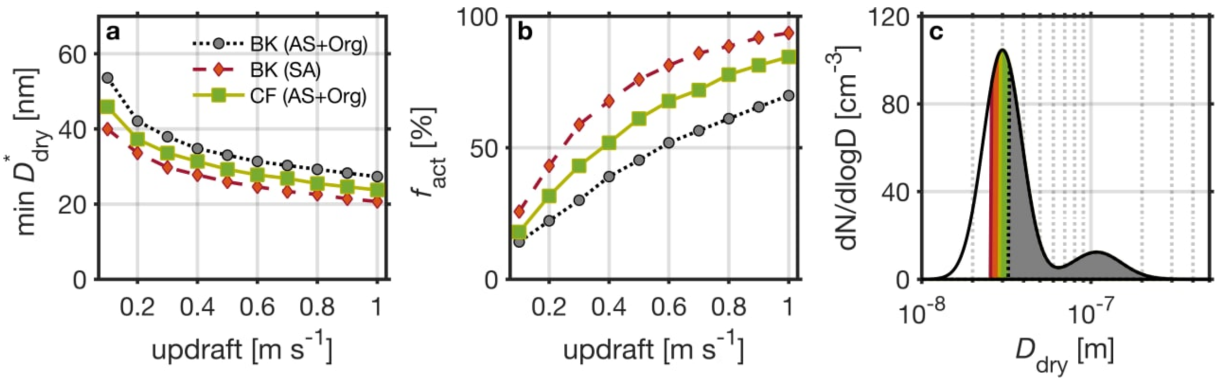

Figure S16. Cloud parcel simulation of the cloud case with high Aitken-mode particle contribution. **a** Smallest activation diameter vs. updraft velocity, **b** activated fraction vs. updraft velocity and **c** size distribution (see Table 1 in main manuscript) with indicated activated fraction at updraft velocity of 0.5 m/s. See text above for details on chemical composition and model assumptions.

Table S5. List of instruments, measured parameters and their temporal resolution.

Instrument	Primary inlet	Parameters	Resolution
DMPS 1 + CPC	GCVI	Particle number size distributions (diameters 10–921 nm), total particle concentrations	9 min, 9 s
DMPS 2 + MCPC	Whole-air	Particle number size distributions (diameters 10–921 nm), total particle concentrations	9 min, 1 s
SMPS + CPCs	Interstitial and whole-air	Particle number size distributions (diameters 18–661 nm), total particle concentrations	3–5 min, 10 s
GCVI	n/a	Visibility	1 s
FSSP-100	n/a	Cloud particle number size distributions (diameters 0.5–47 µm)	1 min
PVM-100	n/a	Liquid water content, cloud particle effective radius	1 s
MAAP	Whole-air	Equivalent black carbon mass concentrations	1 min
AMS	Interstitial and whole-air	Chemical composition of non-refractory material ($< 1\mu$m)	1 min

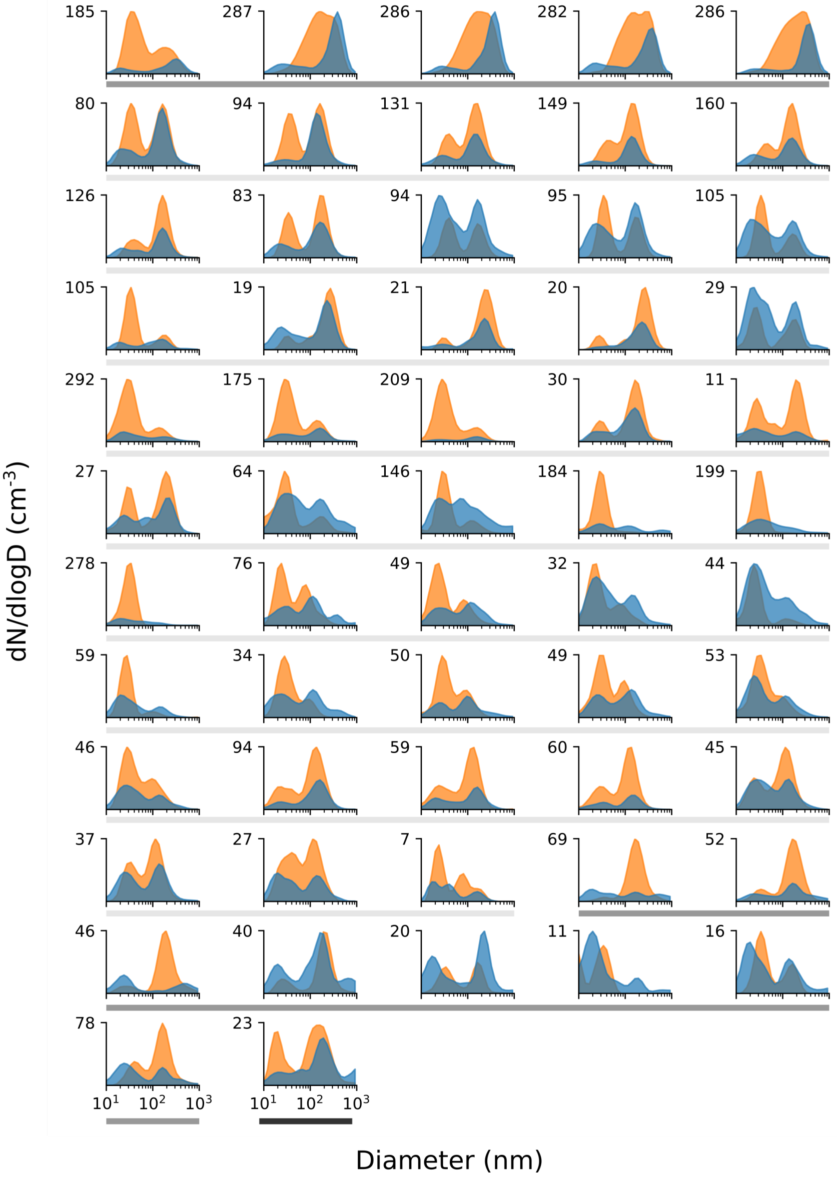

Figure S17. Overview of cloud residual size distributions and corresponding total particle size distributions. Total particle (orange) and cloud droplet residual (blue) size distributions for all observed cloud events. Long events have been divided to show approximately hourly mean size distributions, where each mean contains a minimum of 3 and a maximum of 8 DMPS scans (cf. Fig. 2 in main manuscript). The size distributions have been smoothed by a 5-bin rolling average. Cloud droplet residual size distributions have been multiplied by a constant factor of 17 to account for approximate GCVI transmission efficiency (see Sect. 2 in this SI). Dark gray lines under a panel indicates it is from the MIZ, medium gray from icebreaking, and light gray from the ice drift period. Note the different y-axis scales (scaled to the maximum dN/dlogD value, rounded up to the nearest particle per cubic centimeter).

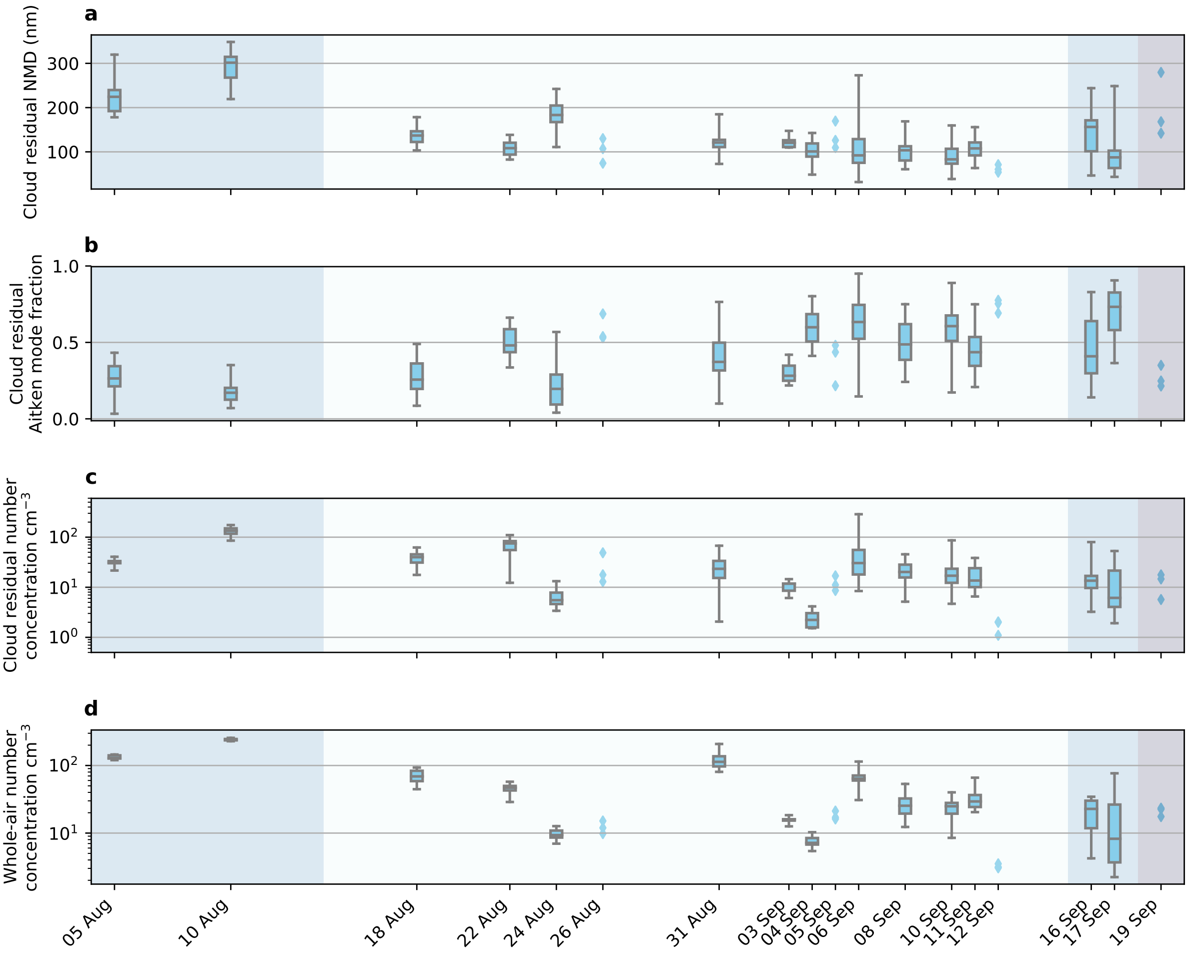

Figure S18. Variability of **a** cloud droplet residual number mean diameter (NMD), **b** cloud droplet residual Aitken mode fraction, **c** cloud droplet residual number concentration, and **d** whole-air particle number concentration during the different cloud events. Cloud events on the same day have been grouped together. When the group includes five or more data points, the data are shown as a box plot where the whiskers extend to the minimum and maximum values. When there are fewer than five data points, the individual points are shown with blue diamond markers. In the background of each panel, the geographical periods from Fig. 3 in the main paper are shaded in: marginal ice zone stations (dark blue), icebreaking periods (medium blue), and the ice drift station (light blue).

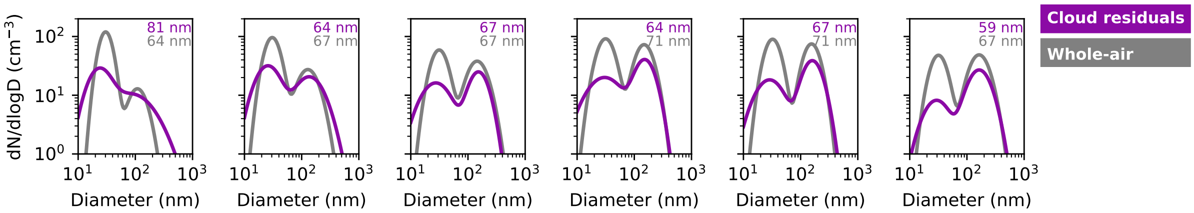

Figure S19. Lognormal fits of the mean cloud residual (purple) and whole-air (gray) particle size distributions for each cloud residual number mean diameter (NMD) bin from Fig. 4 in the main manuscript. The approximate Hoppel minima diameters are also indicated in the figure.

References

Baccarini, A., Karlsson, L., Dommen, J., Duplessis, P., Vüllers, J., Brooks, I. M., ... Schmale, J. (2020). Frequent new particle formation over the high Arctic pack ice by enhanced iodine emissions. *Nature Communications*, *11*(1), 4924. doi: 10.1038/s41467-020-18551-0

Baumgardner, D., Subramanian, R., Twohy, C., Stith, J., & Kok, G. (2008). Scavenging of black carbon by ice crystals over the northern Pacific. *Geophysical Research Letters*, *35*(22), L22815. doi: 10.1029/2008GL035764

Bigg, E. K., & Leck, C. (2001). Cloud-active particles over the central Arctic Ocean. *Journal of Geophysical Research: Atmospheres*, *106*(D23), 32155–32166. doi: 10.1029/1999JD901152

Birch, C. E., Brooks, I. M., Tjernström, M., Shupe, M. D., Mauritsen, T., Sedlar, J., ... Leck, C. (2012). Modelling atmospheric structure, cloud and their response to CCN in the central Arctic: ASCOS case studies. *Atmospheric Chemistry and Physics*, *12*(7), 3419–3435. doi: 10.5194/acp-12-3419-2012

Field, P. R., Lawson, R. P., Brown, P. R. A., Lloyd, G., Westbrook, C., Moisseev, D., ... Sullivan, S. (2016). Chapter 7. Secondary Ice Production - current state of the science and recommendations for the future. *Meteorological Monographs*, *58*, 7.1–7.20. doi: 10.1175/AMSMONOGRAPHS-D-16-0014.1

Graham, R. M., Hudson, S. R., & Maturilli, M. (2019). Improved Performance of ERA5 in Arctic Gateway Relative to Four Global Atmospheric Reanalyses. *Geophysical Research Letters*, *46*(11), 6138–6147. doi: 10.1029/2019GL082781

Hänel, G. (1987). Role of aerosol properties during the condensational stage of cloud: A reinvestigation of numerics and microphysics. *Beitr. Phys. Atmos.*, *60*(3), 321-339.

Karlsson, L., Krejci, R., Koike, M., Ebell, K., & Zieger, P. (2021). A long-term study of

cloud residuals from low-level Arctic clouds. *Atmospheric Chemistry and Physics*, *21*(11), 8933–8959. doi: 10.5194/acp-21-8933-2021

Leck, C., & Svensson, E. (2015). Importance of aerosol composition and mixing state for cloud droplet activation over the Arctic pack ice in summer. *Atmospheric Chemistry and Physics*, *15*(5), 2545–2568. doi: 10.5194/acp-15-2545-2015

Lowe, S. J., Partridge, D. G., Davies, J. F., Wilson, K. R., Topping, D., & Riipinen, I. (2019). Key drivers of cloud response to surface-active organics. *Nature Communications*, *10*(1), 5214. doi: 10.1038/s41467-019-12982-0

Mauritsen, T., Sedlar, J., Tjernström, M., Leck, C., Martin, M., Shupe, M., ... Swietlicki, E. (2011). An Arctic CCN-limited cloud-aerosol regime. *Atmospheric Chemistry and Physics*, *11*(1), 165–173. doi: 10.5194/acp-11-165-2011

Partridge, D. G., Vrugt, J. A., Tunved, P., Ekman, A. M. L., Gorea, D., & Sorooshian, A. (2011). Inverse modeling of cloud-aerosol interactions – part 1: Detailed response surface analysis. *Atmospheric Chemistry and Physics*, *11*(14), 7269–7287. Retrieved from `https://acp.copernicus.org/articles/11/7269/2011/` doi: 10.5194/acp-11-7269-2011

Partridge, D. G., Vrugt, J. A., Tunved, P., Ekman, A. M. L., Struthers, H., & Sorooshian, A. (2012). Inverse modelling of cloud-aerosol interactions – part 2: Sensitivity tests on liquid phase clouds using a markov chain monte carlo based simulation approach. *Atmospheric Chemistry and Physics*, *12*(6), 2823–2847. Retrieved from `https://acp.copernicus.org/articles/12/2823/2012/` doi: 10.5194/acp-12-2823-2012

Porter, G. C. E., Adams, M. P., Brooks, I. M., Ickes, L., Karlsson, L., Leck, C., ... Murray, B. J. (2021). Highly active ice-nucleating particles at the summer North Pole. *Earth and Space Science Open Archive*, 28. doi: 10.1002/essoar.10508073.1

Roelofs, G. (1992). On the drop and aerosol size dependence of aqueous sulfate formation in a continental cumulus cloud. *Atmospheric Environment. Part A. General Topics*, *26*(13), 2309-2321. doi: https://doi.org/10.1016/0960-1686(92)90362-O

Roelofs, G.-J., & Jongen, S. (2004). A model study of the influence of aerosol size and chemical properties on precipitation formation in warm clouds. *Journal of Geophysical Research: Atmospheres*, *109*(D22). doi: https://doi.org/10.1029/2004JD004779

Seinfeld, J. H., & Pandis, S. N. (2016). *Atmospheric Chemistry and Physics : From Air Pollution to Climate Change* (3rd ed.). New York: John Wiley & Sons, Incorporated.

Shingler, T., Dey, S., Sorooshian, A., Brechtel, F. J., Wang, Z., Metcalf, A., ... Seinfeld, J. H. (2012). Characterisation and airborne deployment of a new counterflow virtual impactor inlet. *Atmospheric Measurement Techniques*, *5*(6), 1259–1269. doi: 10.5194/amt-5-1259-2012

Shupe, M. D., Persson, P. O. G., Brooks, I. M., Tjernström, M., Sedlar, J., Mauritsen, T., ... Leck, C. (2013). Cloud and boundary layer interactions over the Arctic sea ice in late summer. *Atmospheric Chemistry and Physics*, *13*(18), 9379–9399. doi: 10.5194/acp-13-9379-2013

Siegel, K., Karlsson, L., Zieger, P., Baccarini, A., Schmale, J., Lawler, M., ... Mohr, C. (2021). Insights into the molecular composition of semi-volatile aerosols in the summertime central arctic ocean using figaero-cims. *Environ. Sci.: Atmos.*, *1*, 161-175. doi: 10.1039/D0EA00023J

Sotiropoulou, G., Sedlar, J., Forbes, R., & Tjernström, M. (2016). Summer Arctic clouds in the ECMWF forecast model: an evaluation of cloud parametrization schemes. *Quarterly Journal of the Royal Meteorological Society*, *142*(694), 387–400. doi: 10.1002/qj.2658

Sumlin, B. J., Heinson, W. R., & Chakrabarty, R. K. (2018). Retrieving the aerosol complex refractive index using PyMieScatt: A Mie computational package with visualization capabilities. *Journal of Quantitative Spectroscopy and Radiative Transfer*, *205*, 127–134. doi: 10.1016/j.jqsrt.2017.10.012

Tjernström, M., Svensson, G., Magnusson, L., Brooks, I. M., Prytherch, J., Vüllers, J., & Young, G. (2021). Central Arctic weather forecasting: Confronting the ECMWF IFS with observations from the Arctic Ocean 2018 expedition. *Quarterly Journal of the Royal Meteorological Society*, *147*(735), 1278–1299. doi: 10.1002/qj.3971

von der Weiden, S.-L., Drewnick, F., & Borrmann, S. (2009). Particle Loss Calculator – a new software tool for the assessment of the performance of aerosol inlet systems. *Atmospheric Measurement Techniques*, *2*(2), 479–494. doi: 10.5194/amt-2-479-2009

Wiedensohler, A., Birmili, W., Nowak, A., Sonntag, A., Weinhold, K., Merkel, M., ... Bastian, S. (2012). Mobility particle size spectrometers: harmonization of technical standards and data structure to facilitate high quality long-term observations of atmospheric particle number size distributions. *Atmospheric Measurement Techniques*, *5*(3), 657–685. doi: 10.5194/amt-5-657-2012

Young, G., Vüllers, J., Achtert, P., Field, P., Day, J., Connor, E. O., ... Iii, R. N. (2021). Evaluating Arctic meteorology modelled with the Unified Model and Integrated Forecasting System. *Atmospheric Chemistry and Physics Discussions*, *2021*, 1—-54. doi: 10.5194/acp-2021-662

Zieger, P., Väisänen, O., Corbin, J. C., Partridge, D. G., Bastelberger, S., Mousavi-Fard, M., ... Salter, M. E. (2017). Revising the hygroscopicity of inorganic sea salt particles. *Nature Communications*, *8*(May). doi: 10.1038/ncomms15883
